# Infrared and Raman spectra of lignin substructures: Coniferyl alcohol, abietin, and coniferyl aldehyde

**DOI:** 10.1002/jrs.5588

**Published:** 2019-04-01

**Authors:** Peter Bock, Notburga Gierlinger

**Affiliations:** ^1^ Institute of Biophysics, Department of Nanobiotechnology University of Natural Resources and Life Sciences Vienna Austria

**Keywords:** abietin, band assignment, coniferyl alcohol, coniferyl aldehyde, lignin

## Abstract

Anatomical and chemical information can be linked by Raman imaging. Behind every pixel of the image is a Raman spectrum, which contains all the information as a molecular fingerprint. Yet to understand the spectra, the bands have to be assigned to components and their molecular structures. Although the lignin distribution is easily tracked in plant tissues, the assignment of the spectra is not good enough to allow in‐depth analysis of the composition. Assignments of three lignin model compounds were derived from polarization measurements and quantum‐chemical computations. Raman spectra of coniferyl alcohol crystals showed orientation dependence, which helped in band assignment. Abietin showed a Raman spectrum that was very similar to the spectrum of coniferyl alcohol, whereas its IR spectrum was very different due to bands of the sugar moiety. The Raman spectrum of coniferyl aldehyde is affected by the crystal order of molecules. All three compounds show much stronger band intensities than unconjugated single aromatic rings, indicating that the bulk of the lignin structure has significantly reduced contribution to Raman band intensities. Therefore, it is possible to highlight certain structures of lignin with Raman spectroscopy, because low amounts of a compound do not necessarily mean weak features in the spectrum.

## 1 INTRODUCTION

Raman imaging of plant materials is a fast and nondestructive technique, which gives insights into the chemistry on a microlevel in context with the anatomical structure. Inhomogeneous samples can be analyzed with little‐to‐no‐sample‐preparation, and a wide range of plant material has been studied.^1^, ^2^, ^3^ Raman images are composed of hundreds to thousands of spectra and are generated by displaying the intensity of one or more bands. Thus based on spectral differences, chemical changes are easily visualized.^3^, ^4^ When it comes to the question how these differences arise, the analysis can get very complicated. This is because vibrational spectroscopy is very sensitive to the structure of a molecule and environment factors like pH, neighboring groups, and so forth can alter the spectrum acquired. This means that even known single structures can display very complicated spectra.^5^

The case is further complicated when it comes to Raman imaging of plants, because the main constituents of plants show a high variability in terms of their chemical structure.^6^, ^7^, ^8^, ^9^ Despite this, Raman imaging can visualize differences in plant chemistry in situ, which is a big advantage. But even more insights will be gained by this method with a better understanding and band assignments of Raman spectra of reference polymers and their components.

One of the main plant polymers is lignin, and it is readily detected by Raman spectroscopy due to its strong signal. Despite its dominance in the spectrum of plant cell walls, relatively less is known about the origin of its bands. The Raman spectra are almost exclusively assigned by the working group of U.P. Agarwal,^10^, ^11^, ^12^, ^13^, ^14^, ^15^ whereas in infrared (IR), Sarkanen,^16^ Hergert,^17^, ^18^ and Faix^19^ mainly contributed to the assignments. This is clearly supported by the sources cited in a recent review,^20^ and it means that a small number of papers serve as the base for an increasing number of studies. Unfortunately most of these studies on band assignments do not show spectra of the reference compounds, although exactly these spectra are needed for further studies and analyses.

In the past decade, a wealth of literature has emerged on the vibrational assignments of molecules with the aid of quantum‐mechanical calculations.^21^, ^22^, ^23^, ^24^, ^25^ Yet this seems not to be reflected in the used assignments; a notable exception is one paper on three aromatic units in lignins.^24^ Furthermore, no comprehensive overview on the vibrational spectra of lignin‐relevant structures is available.

For this study, we chose coniferyl alcohol and its aldehyde. They are precursors of the lignin polymer^6^, ^26^ and therefore are of special interest when tracking lignification in plants. We also included abietin (coniferin), which is the glycosylated transport and storage form^27^, ^28^ of coniferyl alcohol. Despite these substructures being one of the few that can be identified in the spectra, no detailed vibrational analysis of these molecules is available. This is even more of a problem when it comes to understanding lignification in situ, where Raman (and to a lesser extent IR) microscopy are very well‐suited tools. The present study therefore aims to close this gap and help advance the assignment of vibrational spectra of lignin to a next level with detailed information on the structure of lignin by vibrational spectroscopy.

## 2 METHODS

### 2.1 Raman and IR measurements

2‐Methoxy‐4‐methylphenol (purity 98%, order number W267104), abietin (90%, SMB00103), coniferyl alcohol (98%, 223735), coniferyl aldehyde (98%, 382051), (D‐(+)‐glucose (99.5%, G8270), and vanillylidenaceton (98%, 90609) were purchased from Sigma Aldrich (St. Louis, Missouri) and used without further purification. Ethanol (99.5%) was purchased from Merck Millipore (Darmstadt, Germany).

Raman spectra were acquired using a confocal Raman microscope (alpha300RA, WITec, Germany) with a 20x air objective (NA 0.4, Carl Zeiss, Germany). Less than 1 mg of each substance was mounted on an aluminum disk for Raman experiments. For 532‐nm experiments, the sample was excited with a linear polarized Sapphire SF laser (532 nm, Coherent, USA). The scattering was detected with an optic multifiber (50 μm) directed to a spectrometer (UHTS 300, WITec, Germany) equipped with blazed gratings (600 and 1,800 g/mm^−1^, BLZ 500 nm) and a CCD camera (Andor DV401 BV, Belfast, Northern Ireland). 785 nm experiments were conducted on the same instrument, using a linear polarized XTRA II laser (785.008 nm, Toptica Photonics, Germany). The scattering was detected with an optic multifiber (100 nm) directed to a spectrometer (UHTS 300, WITec, Germany) equipped with blazed gratings (600 and 1,200 g/mm^−1^, BLZ 750 nm) and a CCD camera (Andor DU401 DD, Belfast, Northern Ireland). The Raman scattering was collected without polarizers.

Raman spectra/images were acquired with laser powers and integration times to optimize the signal without producing spectral artifacts, as shown in the supplementary library.

Coniferyl alcohol undergoes degradation/polymerization upon laser exposure at 532 nm.^29^ The crystalline powder was therefore soaked with ethanol to allow spectra to be recorded. This was done in the following way. Coniferyl alcohol was mounted on a standard microscopy slide with a cover slip. Ethanol was added at the side and allowed to be soaked in by capillary forces. This process was aided by pressing gently on the cover slip with tweezers. The ethanol began to soak in the powder and dissolved coniferyl alcohol, mainly from the fringe. If the amount of coniferyl alcohol exceeds that of ethanol, the solution becomes saturated and the process slows down. This allows time for recording a Raman image until the ethanol eventually evaporates, which is what happened during the measurement, as found by comparing the visual image before and after.

We recorded four images, two with laser polarization plane 0° and 90°, respectively, and the spectrometer recording radiation in any polarization plane. These images and their spectra are included in this work. Two other images of the same spot were recorded with the laser polarization set to 0° and the spectrometer only recording radiation from 0° or 90°, respectively. Because the latter two showed the same spectra as the former two, but with more noise, they are not included here.

Spectra/pixels of Figures 2, 3, 4, 5, S1, and S2, with good signal‐to‐noise ratios, were averaged, cut (3,785–100 cm^−1^ and baseline corrected (polynomial, 2‐4 iterations) using OPUS 7.0 software (Bruker, Billerica, Massachusetts).

**Figure 1:**
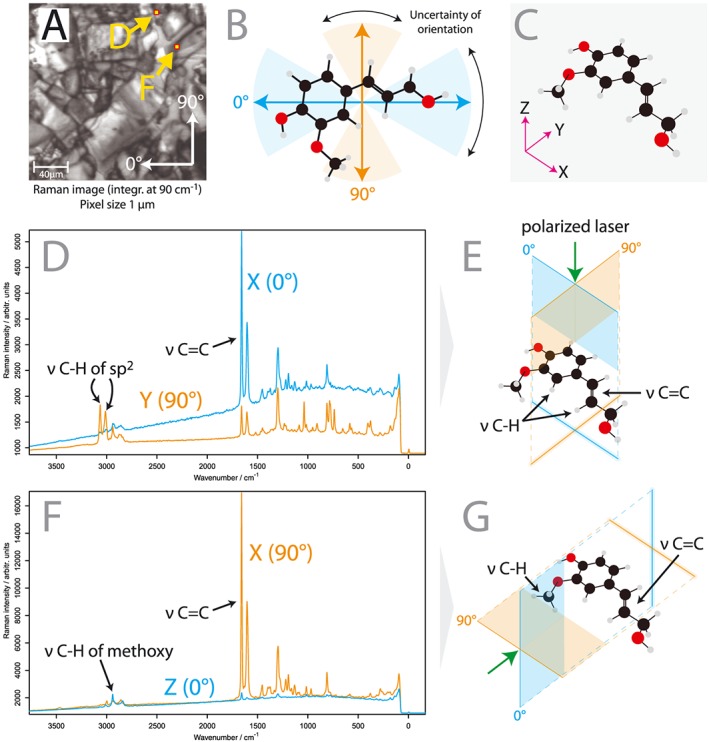
A Raman image of several coniferyl alcohol crystals with laser polarization 0° (A), followed by another one at 90° recorded at the same position (not shown). The spectrometer recorded radiation from any polarization direction. Spectra of the same position differed between the two images; spectra of two pixels—indicated by yellow squares in (A)—are shown in (D) and (F). The difference was attributed to molecular orientation. Although the orientation relative to the laser cannot be estimated exactly—indicated in (B)—the overall molecular orientation can be deduced from the spectra. In (D), one spectrum shows a very strong C=C stretch, whereas the other shows a strong C―H stretch of ring and C=C. If the laser is parallel to the C=C bond, it will give the strongest signal. If the laser is orthogonal to it, the alkene's and one of the ring's C―H bonds are still sufficiently parallel to the laser to give signal. Band assignments support this observation. Similarly, spectra in (F) can be explained by the methoxy group's out‐of‐plane hydrogens and the C=C stretch. On the basis of this, assumed orientations are drawn in (E) and (G). This also suggests that the molecules are rather ordered in the crystal; otherwise, spectra would average out. Combinations of mainly orientation *Y* and *Z* were also found but are not shown [Colour figure can be viewed at wileyonlinelibrary.com]

Spectra of Figures 1 and S3–S6 are raw spectra.

Infrared spectra of pure substances were obtained from a FT‐IR ATR spectrometer (Vertex 70, Bruker, Billerica, Massachusetts) with 16/32 scans. Substances were directly mounted on the ATR unit and measured with the pressure stamp (liquids were measured without stamp). Five measurements were averaged, cut, and baseline corrected using OPUS 7.0 software (Bruker, Billerica, Massachusetts).

### 2.2 NaBH_4_ reduction

The reduction was carried out in an analogous manner as for cinnamyl alcohol, as previously described.^30^ A spruce (*Picea abies*) twig was cut into 1‐cm‐thick disks, which were subsequently cut in half. For the reduction, the pieces were transferred into a flame dried 100‐mL three‐neck‐flask filled with 20‐mL dry MeOH. The flask was put into a crushed ice bath; 776.3 mg NaBH_4_ was added, which resulted in strong bubbling. The flask was then stirred for 24 hr. The wood pieces were then transferred to a beaker and washed with MeOH for 30 min. Finally, the pieces were dried under vacuum to remove excess MeOH. Thin sections of all specimens were cut and measured.

### 2.3 Computational details

The equilibrium geometry and vibrational modes of coniferyl alcohol, abietin, and coniferaldehyde were calculated using the GAMESS package.^31^, ^32^ For coniferyl alcohol and aldehyde, studies on the geometry are available^33^, ^34^ and these geometries were used as starting points for geometry optimization. The SCF‐DFT functional B3LYP with the 6‐311G basis set was used for all calculations. The vibrational modes were visualized with the wxMacMolPlt program.^35^

### 2.4 Vibrational assignment

The assignment of the spectra was done by comparing the spectra with similar compounds in our spectral library, by making use of spectral changes upon modification (i.e., measurement in solid state and in solvents), by polarization Raman measurements, by comparing it with literature results,^5^, ^22^, ^23^, ^36^, ^37^, ^38^, ^39^ and by our own computed results.

## 3 RESULTS AND DISCUSSION

Note: Spectra of all compounds used are also given in the supplementary library, intended to be printed out (A3 format).

### 3.1 Coniferyl alcohol

The Raman images of coniferyl alcohol were acquired in an ethanol environment (see Methods) to acquire the least unchanged spectra. The ethanol evaporated during the measurements, as was noted after 2 hr. The spectra did not show any signs of degradation as previously reported,^29^ so overall, the treatment seemed to be successful.

**Figure 2:**
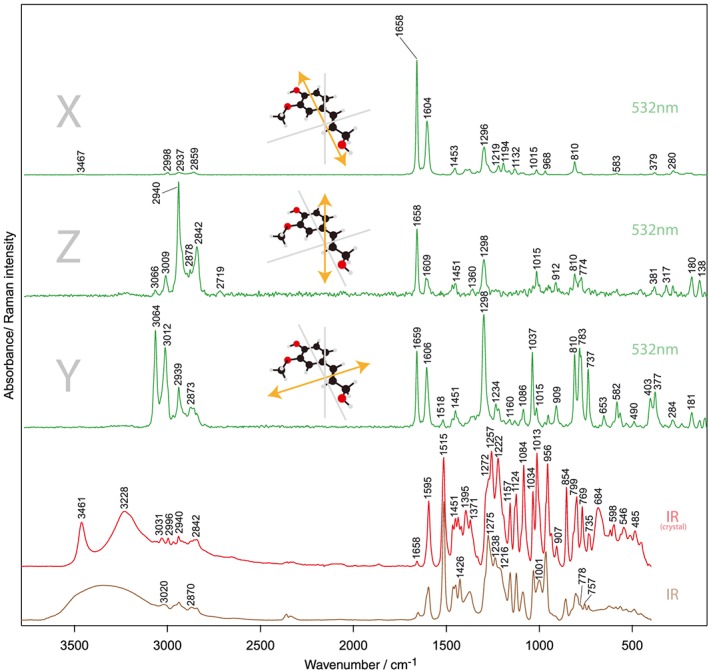
Raman and Infrared spectra of coniferyl alcohol. The Raman spectra shown here are the ones from Figure 1. The orange arrows indicate the laser polarization and show the approximate orientation of the molecules to the laser. The letters X, Y, and Z refer to the cardinal directions of Figure 1. Infrared spectra were obtained from crystalline material and from thin films after ethanol evaporation. Several bands change in intensity, notable being aromatic bands (1,275 and 757 cm^−1^) [Colour figure can be viewed at wileyonlinelibrary.com]

Raman images of coniferyl alcohol crystals yielded strongly differing spectra (Figure 1). Because the purity was high enough (98%), this was attributed to different orientations of the molecules relative to the laser excitation plane. Further indication for this is that on the same pixel, two different spectra could be obtained depending on the polarization direction. This way, three distinctly different spectra were identified. The signal of the C=C stretch is known to decrease upon continued laser irradiation. The spectra in Figure 1d were acquired sequentially by two images, so it could be that the second spectrum—labeled Y(90°)—shows already degraded coniferyl alcohol. The spectra shown in Figure 1f have the reverse order, the more intense spectrum—labeled X(90°)—was recorded after the low intensity spectrum, Z(0°). This suggests that no damage occurred and that the different spectra are therefore linked to different orientations of the molecules. To our knowledge, no studies on the crystal structure of coniferyl alcohol exist, so we do not know the exact orientation of the molecules in the crystal. However, it is clear from the Raman spectra that there must be some overall orientation; otherwise, the spectra would not differ that strongly. In such a case, the spectrum would probably look always like Figure 2, ***X***, because ring and C=C give the strongest signal, as observed for the similar liquid isoeugenol.

The orientation of coniferyl alcohol in the crystal is also reflected in its IR spectra by several shoulders/splits. We were able to recrystallize this substance directly on the powder ATR unit by adding a drop of ethanol, which dissolved the crystals. Once ethanol evaporated, a thin film remained, and its spectra showed some band and intensity shifts compared with the crystal state (Figure 2).

Having oriented molecules in Raman means that the band assignment is facilitated, especially for simple planar molecules like coniferyl alcohol. The following band assignment is therefore based on this evidence as well as on classical textbooks on vibrational assignments^5^, ^36^, ^37^ and the literature published previously on similar compounds.^22^, ^23^, ^24^

The bands will be discussed in decreasing wavenumber order, and the Raman wavenumbers refer to 532‐nm spectra. All spectra are shown in Figure 2. Detailed sheets with assignment as well as spectra of other discussed compounds can be found in the supplementary material.

In IR (Figure 2), the OH stretch comes at 3,461 and 3,228 cm^−1^, with the former band being sharper. The aryl‐OH group is expected to be held in plane of the aromatic ring with less rotational degrees of freedom, and this should produce a sharp band, 3,461 cm^−1^ is therefore assigned to the OH group on the ring (see also the discussion below on the torsion of this group). The other OH group should have less restrictions and be able to participate freely in H‐ bonding, so the broad band at 3,228 cm^−1^ is assigned to the OH stretch of the aliphatic chain. For the next bands, we make use of the different polarization spectra recorded. The CH stretches in conjugated aromatic rings are often very weak, if these groups are not abundant and therefore hard to see if other vibrations have much stronger intensity. This is the case for the Raman spectrum labeled as (orientation) ***X***. Our interpretation is that Coniferyl alcohol is orientated with its longitudinal (*x*) axis parallel to the electric field of the laser.

The C=C stretch (1,658 cm^−1^) and the ring stretch Φ8b (1,604 cm^−1^, ring vibration labeled after Wilson/Varsanyi^37^, ^40^) are the strongest bands in Raman orientation ***X*** (Figure 2, *X*), because these vibrations are also along the *x* axis. In orientation ***Y***, the spectrum looks drastically different, and we explain this with the laser‐oriented perpendicularly to *x* axis of the molecule. This means that the change in polarizability of the C=C bond would be perpendicular to this bond, and also the Raman band would be weak. Conversely, modes, which change the polarizability along the molecule's *y* axis should be strong, and this is the case for CH stretches of the aromatic ring (Φ2 at 3,064 cm^−1^) and of the double bond (3,012 cm^−1^). The latter assignment was retrieved from laser‐irradiation studies performed on coniferyl alcohol.^29^ The band at 2,940 cm^−1^ is assigned to the asymmetric CH stretch of the methoxy group, and in some spectra (Figure 2, ***Z***), this mode was the strongest band.

Because geometry measurements and calculations^41^ show the molecule to be planar, the only C―H bonds, which point out of the plane, are those of the methoxy group and the methylene group. It is well‐known that the asymmetric CH stretch of methyl groups comes around 2,960 cm^−1^,^5^, ^36^ so this band is assigned to the asymmetric CH stretch of the methoxy group. Its symmetric stretch is assigned to 2,842 cm^−1^ for the same reason, although it could also be from the symmetric methylene stretch. Because of this, the third Raman spectrum (Figure 2, ***Z***) was presumably acquired with the laser being mainly aligned with the molecule's *z* axis.

The C=C stretch is seen as strongest band at 1,658 cm^−1^ (Figure 2, ***X***) as mentioned before. Interestingly, its wavenumber is found to be unchanged from the reference value (1,660 cm^−1^ in fatty acids), although its intensity is enhanced. This means that despite the increase in polarizability, the electrons are fairly localized in this bond, something that we will later see to be changed in the aldehyde. Almost no dipole moment change is associated with this vibration, so its IR intensity is very low. The members of Vibration 8 (one of the ring stretches) are found at 1,604 cm^−1^ (8b) and 1,595 cm^−1^ (8a), respectively. Ring stretch 19b is very strong in the IR at 1,515 cm^−1^, but only weak in Raman, owing to its symmetry. CH bending of the methoxy and the methylene groups is seen as several bands from 1,465–1,352 cm^−1^. Among these bands, ring stretch 19a is assigned to the band at 1,425 cm^−1^. Another ring stretch, 14, is assigned to 1,367 cm^−1^, based on DFT calculations of related compounds.^22^

The third strong band of coniferyl alcohol (strongest band in orientation ***Y***) is the CH bend of the C=C. Counter motion of the carbons could mimic the C=C stretch mode, which accounts for the rather strong intensity, as assumed by the displacement pattern of our DFT analysis. This vibration is coupled to the CH in‐phase bending of the ring (Φ3, seen at 1,296 cm^−1^, no IR counterpart). The other combination has less Raman intensity but increased IR activity and is visible as a shoulder at 1,279 cm^−1^.

In vibrational spectroscopy of lignin, diagnostic bands of guaiacyl (4‐hydroxy‐3‐methoxyphenyl) units are called “G‐bands.”^19^, ^42^, ^43^, ^44^ One of these bands is caused by the in‐phase substituent stretching and labeled as Φ7a. In coniferyl alcohol, this band comes at 1,257 cm^−1^. The actual Raman activity seems to be dependent on the involvement of the ring carbons and the attached carbon substituent. If the normal coordinate is centered on the in‐phase stretching of the two C―O oscillators, then the Raman signal is rather weak due to the strong dipole moment associated with this mode. If the normal coordinate involves the ring carbons and the third substituent, then the mode has more breathing character and therefore gains activity in the Raman spectrum. Overall, this makes Φ7a a good group frequency in IR (compare Figure S1) but not in Raman. The neighboring strong band is attributed to another form of substituent stretching (Φ13, 1222 cm^‐1^), which is also intense in the IR and weak in Raman. G rings often display both of these IR bands clearly (see also Figure S1), which can be used for diagnostic purposes. Rocking of the CH_3_ group is found at 1,194 cm^−1^ with medium Raman intensity. Two medium strong IR bands come next; they are assigned to out‐of‐phase substituent stretching (Φ7b, 1,157 cm^−1^) and ring‐CH bending (Φ15, 1,132 and 1,124 cm^−1^). The C―C stretch in the aliphatic chain is assigned to 1,084 cm^−1^ by comparison with similar molecules (see Figure S1 and supplementary information) in accordance with cinnamyl alcohol.^23^ The C―O stretch including the sp^3^‐carbon of the methoxy group is only seen in orientation ***Y***, which is another indication that here, the laser is parallel to the molecule's y axis as this bond is in plane with the ring. Its corresponding IR band (1,034 cm^−1^) is of medium intensity. The neighboring band (1,013 cm^−1^) is assigned to the C―O stretch of the propenol moiety.

The CH wag of C=C is found as a strong IR band at 956 cm^−1^(Figure 2). Reduced symmetry of the molecule results in some Raman activity, which has been demonstrated to be useful for distinguishing pinosylvins.^45^ Out‐of‐phase‐CH‐out‐of‐plane bending (Φ10a) is assigned to the IR band at 936 cm^−1^. The symmetric C―O―C stretch of the methoxy substituent is seen at 915 cm^−1^ (IR). The following two bands are again CH‐out‐of‐plane bendings. The calculation shows involvement of the ethenyl hydrogen in both modes, which are described as Φ10b (854 cm^−1^) and Φ11 (826 cm^−1^).

The next bands (810–737 cm^−1^) deserve more detailed discussion because here, important modes appear in asym‐trisubstitution—these are Φ1, Φ12, and Φ4. Mode 1 is totally symmetric and normally gives the strongest Raman band. However, if the aromatic ring is conjugated, one member of Vibration 8 normally becomes the strongest ring mode, and Φ1 remains weak. In some symmetry groups (C_2v_, D_3h_), Φ12 can become very strong and replaces Φ1 as the strongest mode. Both modes interact with substituent stretches in the case of asym‐trisubstitution, and as they cannot be distinguished on theoretical grounds, it is convention to assign the less loaded oscillating triangle of the David's star to Vibration 12.^37^ Mode 4 is ring puckering and often assigned to weak bands.^22^, ^24^

Coniferyl alcohol shows three bands in this region; these are 810, 783, and 737 cm^−1^; all of them are split in Raman orientation ***Y***. In orientation ***X***, only 810 cm^−1^ appears and is assigned to the light triangle, because in 2,6‐dimethoxy‐4‐methylphenol^24^ this band remains. The computation shows involvement of the C―OH bond, which could explain the orientation dependence. In contrast, the picture for the other triangle is not so clear, as some G rings show only one other strong band (2‐methoxy‐4‐methylphenol; watermelon ketone), whereas others have two medium bands (vanillyl alcohol, 3,4‐dimethoxybenzyl alcohol). In coniferyl alcohol, 783 and 737 cm^−1^ are intense in orientation ***Y***, so in‐plane character is assumed for both. In Φ12 and Φ4, each ring carbon moves out‐of‐phase with respect to its neighbors,^37^ therefore coupling of these modes should be possible. This would mean that each coupling product would have partial in‐plane‐bending character and which could explain the activity of 783 and 737 cm^−1^. The extent of coupling of course depends on the exact ring configuration, and therefore the intensity patterns of these two bands generally found in G rings will vary. To make this coupling formally possible, the assignment order has to be reversed, and we therefore assign 810 cm^−1^ to mode 1 and 783 and 737 cm^−1^ to the coupling products (linear combinations) of modes 12 and 4, respectively.

The broad absorption in IR (crystal) at 684 cm^−1^ is ascribed to hindered rotation (torsion) of H‐bonded OH groups^38^, ^46^, ^47^ and therefore assigned to the phenolic OH group, which seems to be held in plane of the ring (as suggested by the DFT calculation). Abietin (no phenolic OH, see below) does not have this band, which supports the assignment. This band (and the associated stretch of this group) disappears in the ethanol‐thin‐film spectrum—the strict order of the crystal is lost, resulting in more rotational freedom of this group. Bands lower than 650 cm^−1^ are difficult to assign, because only weak bands are observed here in the IR and they differ between both IR spectra.

### 3.2 Abietin (coniferin)

Abietin is the glycosylated form of coniferyl alcohol.

**Figure 3:**
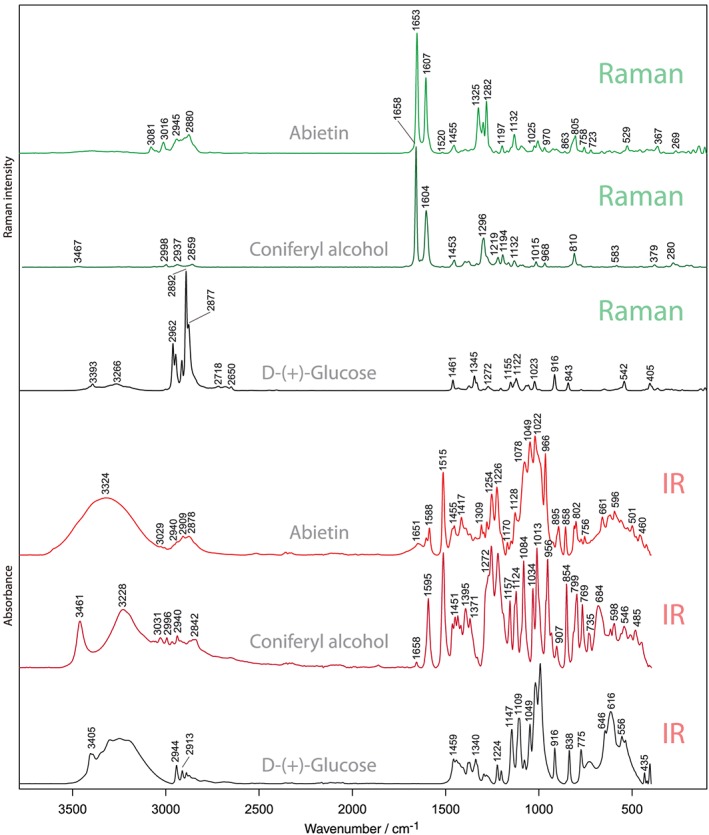
Raman and infrared (IR) spectra of abietin, coniferyl alcohol, and glucose. The latter represent the aromatic and the sugar part of abietin. It is apparent that the Raman spectrum mainly represents the aromatic part. Only the broad saturated C―H stretch (3,000–2,800 cm^−1^) indicates the presence of glucose. In comparison, the IR spectrum of abietin shows a typical band complex of carbohydrates (1,200–900 cm^−1^) beside strong aromatic bands (1,515, 1,254, and 1,226 cm^−1^). It is therefore much easier to deduce the presence of glucose in IR than in Raman [Colour figure can be viewed at wileyonlinelibrary.com]

When comparing the Raman spectra of abietin and coniferyl alcohol (Figure 3), their similarity is apparent. This is a good example showing that in Raman, the conjugated aromatic nucleus produces stronger signal than aliphatic structures and therefore is able to mask them. None of the strong bands comes from the sugar moiety; in fact, its presence can best be concluded by the CH stretches below 3,000 cm^−1^. By contrast, the IR spectra (Figure 3) appear different at first glance and the presence of carbohydrates can be assumed by the typical band complex between 1,200 and 900 cm^−1^.

In IR (Figure 3), the OH stretch is centered on 3,324 cm^−1^ and broad due to a lot of different OH groups in different rotational configurations and participating in various H bonds. CH stretching of unsaturated carbons is visible in the Raman at 3,081 (Φ2) and 3,016 cm^−1^ (C=C). The CH stretching of saturated carbons fills the whole range from 3,000 to 2,800 cm^−1^.

The strongest Raman band is again the C=C at 1,654 cm^−1^, flanked by the aromatic ring stretch (Φ8b, 1,607 cm^−1^) as the second strongest band. Another ring stretch (Φ19b) comes at 1,515 cm^−1^ and is very strong in the infrared. CH bending comes next and produces various bands in the IR (1,485–1,365 cm^−1^), with another ring stretch arising over them (Φ19a, 1,417 cm^−1^). The next three bands are all strong in Raman. They are all assigned to various forms of CH bending, and the modes are likely to couple with each other. The higher wavenumber bands probably resemble more the motions of the hydrogens of the ethenyl group (1,325 and 1,299 cm^−1^), whereas 1,282 cm^−1^ can be described as a ring mode (Φ3). The calculation also shows aliphatic hydrogens of the sugar to be involved in these motions, but their intensity contribution is assumed to be low because of the enhancement of the aromatic modes. Two ring substituent modes (Φ7a and Φ13) are observed as strong IR bands at 1,254 and 1,226 cm^−1^.

The IR bands at 1,194 and 1,170 cm^−1^ are assigned to OH bending and to C―H rocking of the methoxy group. By comparison with other similar molecules (see also Figure S1), substituent stretching Φ7b and C―H bending Φ 9b are assigned to 1,150 and 1,128 cm^−1^, respectively.

The strongest IR bands of abietin are caused by aliphatic C―O and C―C stretchings. By comparing the IR spectrum of pristine D(+)‐glucose, the IR bands at 1,049, 1,022 and 994 cm^−1^ are assigned to the sugar moiety, whereas the bands at 1,078 and 1,005 cm^−1^ are assigned to the 3‐hydroxy‐prop‐1‐en‐1‐yl moiety of the molecule. The fact that aromatic rings participating in conjugation produce much stronger Raman signal^48^, ^49^, ^50^ is also shown by the observation that despite the strong IR fingerprint of the sugar, its Raman bands are insignificant.

CH wagging of the ethenyl group is observed as a strong band at 966 cm^−1^ in the IR; the bands next to it (926 cm^−1^) is probably caused by a CH ring mode. On the basis of coniferyl alcohol, the band at 904 cm^−1^ is assigned to the symmetric C―O―C stretch of the methoxy group. The lone‐H‐wag of the ring is seen at 858 cm^−1^ (Φ10b) and the umbrella mode at 812 cm^−1^ (Φ11).

The Raman band at 824 cm^−1^ has no IR counterpart, but its origin remains unclear. Ring stretching (Φ1) is assigned to the band at 802 cm^−1^. The IR band at 775 cm^−1^ is assigned to a mode of glucose; this is supported by the fact that this band is not enhanced in the Raman spectrum unlike the adjacent ring modes.

The bands at 756 and 720 cm^−1^ are described as ring modes. Their assignment follows the explanation for coniferyl alcohol.

The remaining bands are difficult to assign. Even by comparison of the spectra, it remains unclear whether a band arises from the sugar or from the aromatic part. Both glucose and coniferyl alcohol have coinciding bands in this region, and also the aromatic enhancement cannot be exploited because no strong bands of coniferyl alcohol exist in this part of the spectrum.

### 3.3 Coniferyl aldehyde

**Figure 4:**
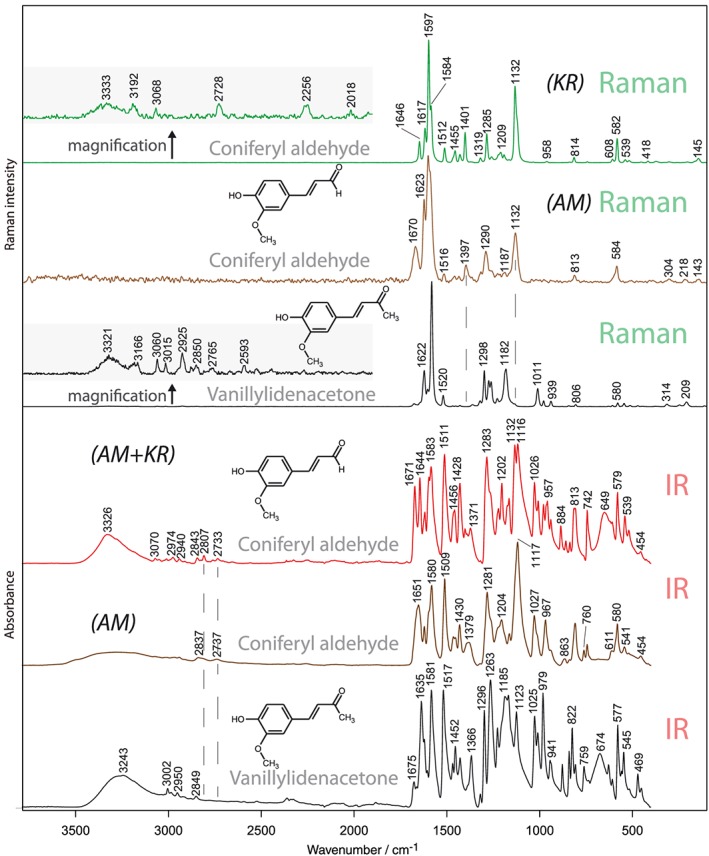
Raman and Infrared (IR) spectra of coniferyl aldehyde and vanillylidenacetone. The latter is shown for comparison to highlight aldehyde‐specific Raman bands (1,401 and 1,132 cm^−1^). The spectrum named KR was obtained from a crystal, AM represents an amorphous phase (cooled down after melting). The IR spectra of coniferyl aldehyde are shown for the mixture of amorphous phase with crystalline phase (AM + KR, compound as received). Band splitting is removed in the spectrum of the cooled melt (AM). The IR spectra of the aldehyde show typical Bohlmann bands (dashed line). Aldehyde and ketone are much harder to distinguish in IR than in Raman [Colour figure can be viewed at wileyonlinelibrary.com]

Coniferyl aldehyde exists in two different crystalline forms, as reported by Stomberg et al.^34^ The sample purchased from Sigma Aldrich (Austria) not only consists mostly of an amorphous, orange‐to‐brown powder (in the following *AM*) but also of small (~50 μm) crystals (termed *KR*). These displayed Raman spectra with sharp lines, practically free of background (Figure 4), which can be related to the high order in the crystal.^51^ Unlike for coniferyl alcohol, changing the laser polarization did not yield different spectra in terms of different wavenumbers (the intensity changed though, which points to a weak overall orientation). Additionally, coniferyl aldehyde was measured as cooled melt to estimate the influence of the crystal order on the vibrational spectra.

The overall impression of the Raman spectrum is typical for small organic compounds, where a phenyl ring is conjugated to a carbon double bond. Even on crystalline spectra, the CH stretching modes can only be seen upon strong magnification of the respective region—also, this is often seen when working with conjugated aromatic molecules.

Due to the limitations of the equipment used for acquisition of IR spectra, it was not possible to acquire spectra of crystals. The IR spectrum of the powder therefore represents a mixture of at least two species (*AM* and *KR*). Cooling molten coniferyl aldehyde resulted in a highly viscous liquid at room temperature, which yielded slightly different spectra (*AM*), as shown in Figure 4. The same spectra have been obtained from the powder dissolved in acetone once the solvent had evaporated. This should represent the *AM* configuration.

In the following section, mainly the crystal Raman spectrum (*KR*) and the powder IR spectrum (*AM + KR*) will be discussed with reference to the other spectra if necessary for explanation. The interpretation of the infrared spectrum is complicated by the fact that there are at least two different species of coniferyl aldehyde present, which results in a lot of split bands.

The OH stretch is observed at 3,326 cm^−1^ as a broad band in IR. The corresponding Raman band at 3,334 cm^−1^ is very weak. The band at 3,192 cm^−1^ is assigned to the overtone of the quadrant ring stretching (2 × 1,597 = 3,194), in accordance with the literature.^52^, ^53^

Ring CH stretching is observed at 3,068 (Raman, probably Φ2) and 3,049 cm^−1^. The CH stretching of the ethenyl group is assigned to the IR band at 3,009 and the asymmetric stretching of the methyl group's hydrogens to the bands at 2,974 and 2,940 cm.^−1^ The weak, but distinctive Bohlmann bands^36^, ^54^, ^55^ appear at 2,807 and 2,733 cm^−1^ in the IR. They are split due to interaction with the bending mode of this bond,^36^ The weak Raman band at 2,728 cm^−1^, however, might be a combination band of Φ8b with the aldehyde C―C stretch (1,597 + 1,132 = 2,729).

**Figure 5:**
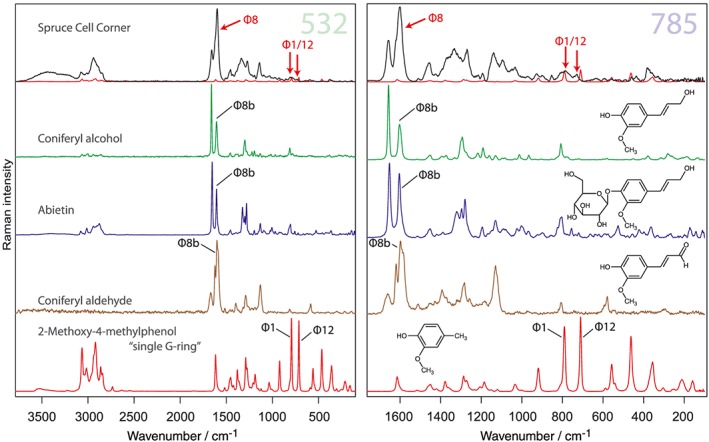
Raman spectra of a cell corner of spruce, of all three compounds discussed herein and of 2‐methoxy‐4‐methylphenol. The latter serves as a model compound for the lignin polymer (G ring). The left part of the figure shows spectra excited by 532 nm and the right part spectra of 785 nm. Spectra of coniferyl alcohol, aldehyde, and abietin are very similar to the lignin spectrum in the cell corner, regardless of the excitation wavelength. On the other hand, the strongest bands of the “single G ring” (Φ1, Φ12) are very weak in the cell corner spectrum (red arrows). This means that the cell corner spectrum mainly shows conjugated aromatic substructures (compare Φ8), whereas unconjugated rings, although in majority, can hardly be seen in the Raman spectrum. Note that the “single G ring” is also drawn in the cell corner spectrum [Colour figure can be viewed at wileyonlinelibrary.com]

The band at 2,256 cm^−1^ is assigned to the first overtone (2 × 1,132 = 2,264) of the C―C stretch of the C=C―C=O group (see magnified spectrum in Figure 5).

Stretching of the carbonyl group is found at 1,671 and 1,644 cm^−1^ in the powder and as a broader band centered at 1,651 cm^−1^ in the cooled melt (*AM*). These spectral differences have already been reported^34^ without further comment. Regarding our spectra, we have already noted that a single crystalline form could only be probed by Raman, whereas the IR spectrum of the powder still represents a mixture. By comparing carbonyl frequencies of various molecules with a C=C―C=O moiety, we find that the C=O stretch is found close to 1,670 cm^−1^ (1R‐(‐)‐myrtenal 1675, cinnamaldehyde 1669, o‐methoxycinnamaldehyde 1671, 4‐acetoxy‐3‐methoxycinnamaldehyde 1673). In none of these compounds, a hydrogen bond to the carbonyl can be established that is why we regard 1,670 as the unperturbed wavenumber of (phenyl)propenals and therefore assign the 1,671 cm^−1^ of coniferyl aldehyde to a carbonyl group not participating in H bonding. However, in the crystal structure described by Stomberg et al.,^34^ the hydroxyl group on the ring is hydrogen‐bonded to the carbonyl group. Hydrogen‐bonding will further reduce the stretching wavenumber^5^, ^36^; therefore, we assign the IR band at 1,644 cm^−1^ to the H‐bonded C=O stretch. Due to conjugation, this group is also activated in the Raman spectrum where it is observed at 1,646 cm^−1^ (crystal).

Upon melting, more configurations of this group are possible, indicated by a broadening and wavenumber‐shifting of this band (Raman 1,670 cm^−1^; indicating less hydrogen bonding based on the wavenumber considerations above). Coniferyl aldehyde does not recrystallize upon cooling, and a single broad band is also found in the IR (1,651 cm^−1^). Published work questions this assignment,^56^ but based on our analysis, we reject this proposal and support the assignments made by Agarwal et al.^10^, ^12^ that the carbonyl stretch has a higher wavenumber than the C=C stretch, which is observed at 1,617 cm^−1^. The effect of conjugation with the carbonyl group can be observed in the infrared spectrum in comparison with coniferyl alcohol/abietin—it is much stronger for the aldehyde. The same is true for the quadrant ring stretches (1,595 cm^−1^, 8b, and 1,583 cm^−1^, 8a), which are of similar intensity to the half‐circle ring stretch 19b at 1,511 cm^−1^.

Bending of the methoxy group's hydrogens is seen at 1,456 cm^−1^. It consists of two bands, with a split seen in the IR spectrum of the melt. Because the methoxy group is held in the plane of the ring, the asymmetric bending is no longer degenerated, and two bands are expected. Φ19a is found at 1,428 cm^−1^. Bending of the lone hydrogen of the aldehyde group produces a sharp Raman band in the crystal spectrum (1,401 cm^−1^). Kekulé‐stretching coupled with OH bending^57^ is assigned to the IR band at 1,371 cm^−1^. A CH‐bending mode (Φ3) is assigned to the weak band at 1,318 cm^−1^. The “G band” (see coniferyl alcohol) occurs at 1,285 cm^−1^ in Raman, the IR counterpart is split (1,283 and 1264 cm^−1^). In this case, the splitting is explained as an intramolecular coupling, as there is no difference between the IR spectra observed. Interaction of a ring mode (Φ7a) with CH‐in‐plane‐bending of sp^2^‐carbons is therefore taken as the assignment. The IR band at 1,220 cm^−1^ is assigned to another ring mode (Φ13). OH bending coupled with CH_3_ rocking is computed to be responsible for the next bands (1,202 and 1,192 cm^−1^). The IR band at 1,162 cm^−1^ (split in crystal form) is correlated with substituent‐stretching (Φ7b).

The strongest band in IR (1,132 cm^−1^) is attributed to the C―C stretch of the aldehyde group. In the Raman spectrum, it is the second strongest band. The IR band is split (1,132 and 1,116 cm^−1^) in two equal intense bands; the Raman band (1,132 cm^−1^) shows a shoulder at 1,123 cm^−1^ with half intensity. On the basis of the discussion of the carbonyl group, one could argue that the IR spectrum shows two C=O peaks; therefore, also two C―C stretches would be expected. Because both doublets have intensity ratios of 1:1, this seems reasonable. In the IR spectrum of the *AM*, 1,132 cm^−1^ decreased to a shoulder of 1,116 cm^−1^. Moreover, the skew towards lower wavenumber decreased. However, the Raman spectrum of the crystal also shows two peaks (1,132 with shoulder at 1,123 cm^−1^). Due to one C=O species present in the crystal, a split due to different C=O groups is therefore ruled out. Fermi resonance of the C―C stretch with the C=O bend is ruled out as well (a detailed discussion on this can be found in the supplementary information together with Figure S2). The 1,132‐band is therefore assigned to the C―C stretch of the aldehyde, whereas the 1,116‐band is interpreted as a CH‐bending mode (Φ9b) with contribution of the C―C stretch.

All the following bands are weak in the Raman spectrum; the description will center only on the IR bands. The single C―O stretch of methoxy group is seen at 1,026 cm^−1^. The C=C―C=O group has three hydrogens, so there will be three out‐of‐plane modes. The in‐phase‐wag is assigned to 1,006 cm^−1^, the trans‐wag of the C=C countered by the aldehyde's H to 978 cm^−1^, and the third combination couples with the lone‐H‐wag of the ring to give two bands at 884 and 857 cm^−1^, only the latter is found in the spectrum of *KR*. Out‐of‐phase‐wagging of the two vicinal hydrogens (Φ10a) is assigned to 938 cm^−1^. The band at 957 cm^−1^ is interpreted as the symmetric C―O stretch of the methoxy group (see supplementary information).

The symmetric ring stretch (Φ1) is one of the few stronger Raman bands in this region, and there is an intensity difference between the crystalline and the amorphous IR spectra. The crystal IR band is split (813 and 806 cm^−1^). The Raman spectrum only represents *KR*, so the other band is attributed to Φ1 of *AM*.

The weak IR band at 763 cm^−1^ is attributed to ring puckering (Φ4) and the medium band at 742 cm^−1^ to the bending of the heavy loaded triangle (Φ12). The shoulder (731 cm^−1^) is also attributed to Φ12 of *KR*.

The broad absorption at 650 cm^−1^ is the OH‐torsion band of *KR*. It is absent in *AM*. In comparison with coniferyl alcohol, its wavenumber is lower, which implies that the H bond is weaker in the aldeyhde.^38^

Most of the molecules we measured having an C=C―C=O group show an IR band in the region 590–560 cm^−1^, which is attributed to the C=O bend. Coniferyl aldehyde shows this band at 579 cm^−1^, but there is also a medium–strong Raman line at the same position (582 cm^−1^), indicating an aromatic band. Depending on the calculation, ring mode 6a is coupled to the bending mode, and a study on benzaldehyde has also the carbonyl bending and ring bending coupled.^15^ The same study^15^ assigned the C=O bend of vanillin to 588 cm^−1^. Considering all this, we assign this band to a combination of both modes.

### 3.4 Distinguishing substructures in cell wall spectra

#### 3.4.1 Raman intensities

Despite the fact that lignin contains relative low amounts of coniferyl alcohol (2 %) and aldehyde (4 %),^58^ they can be detected very well in the Raman spectrum of lignin. The reason is enhancement of the signal by extended electron clouds and charge transfers.^50^, ^59^, ^60^, ^61^, ^62^

Intensities are an important part of the interpretation of Raman spectra. We have to distinguish between intramolecular intensity ratios, that is, band ratios in the spectrum of a given molecule, and intermolecular intensity ratios, which derive from different Raman cross‐sections. The molecules studied in our work serve as good examples, because intensity is the basis for their easy detection in the Raman spectrum.

Although preresonance enhancement is often used for explaining strong signal of lignin substructures, it is not the only cause for strong signal.

This so‐called *conjugation effect* was noted decades ago^48^ and is very visible in the Raman spectra presented here (compare Figures 2, 3, 4). It has been explained that the change in polarizability extends beyond the normal coordinate of the vibration^50^ and is therefore independent from the excitation wavelength (compare the shape of the spectra given in the supplementary library).

Another proposed effect takes the bond‐length‐alternation‐oscillation into account, which occurs in charge‐transfer systems and is called *Я‐effect*.^61^, ^63^ Coniferyl aldehyde is such a charge‐transfer molecule.^64^, ^65^

The result is that certain lignin substructures like coniferyl aldehyde can be observed very well in the Raman spectrum of lignin, despite their total amount being very low (<5%).^58^ This is also seen experimentally (see Figure S3), where spectra recorded with the same laser power and integration time show drastic differences. This implies that the majority of the lignin polymer structures contribute weakly in untreated samples. Our measurements suggest that this can also not be amended by increasing the excitation wavelength, because the Raman cross section is an intrinsic property of the molecule (compare Figures S4 and S5). Therefore, even FT‐Raman measurements^11^ show this behavior. Figure 5 compares the lignin spectrum of spruce against the three molecules in this study. A single G‐lignin ring is also shown. Because most of the aromatic rings in lignin are unconjugated, their spectra should be similar to that of the single ring. The single ring is a valid comparison, because we expect all of the aromatic rings in lignin to be effectively decoupled from each other. In unconjugated rings, normally Mode 1 or 12 cause the strongest bands (see Figure 5 “single G ring”). Upon conjugation, Mode 8 frequently becomes the strongest band instead; this is also the case for coniferyl alcohol, its sugar and its aldehyde. Because Raman spectra of untreated lignin look like that shown in Figure 5, it is clear that the spectrum is mainly caused by strong (i.e., conjugated) scatterers and that the bulk of the polymer (unconjugated rings) is underrepresented (compare the intensity of Φ1 against Φ8). This is not a new finding (compare the publications of Agarwal et al. cited herein) but has to be emphasized again, because strictly speaking, the Raman spectrum of lignin is dominated by some substructures, and the remaining structures contribute only weakly.

#### 3.4.2 Raman wavenumbers

Coniferyl alcohol has three prominent bands in the Raman spectrum, but only two of them are characteristic for it, because the third being the aromatic ring stretch, which is shared by all conjugated arenes. The C―H bend at 1,296 is not useful for lignin spectra, because it falls into a band complex, which is constituted of C―X stretching and C―H bending modes of many aromatic compounds. The band at 1,660 is the only one from which the existence of coniferyl alcohols can be judged (if other cinnamyl alcohols can be excluded), although it should be kept in mind that cinnamyl aldehydes also contribute to this band. However, as has been noted very recently, the presence of cinnamyl alcohols might be estimated by controlled laser degradation/polymerization experiments, which will only affect the alcohol.^29^

Abietin only differs in that it has two additional strong Raman bands than coniferyl alcohol, both of which come into the aforementioned band at 1,380–1,250 cm^−1^. Therefore a judgment based only on the Raman spectrum is not recommended.

Among these three compounds, coniferyl aldehyde is the easiest to identify, because it has the most diagnostic bands—1,620, 1,400, and 1,135 cm^−1^. Other candidates for this band are not present (cinnamyl ketones), or their C=C stretches are upshifted (cinnamic acids), so at present, this band is only known to derive from coniferyl/sinapinaldehyde. The 1,135‐band suffers a great intensity loss upon NaBH_4_ reduction (which reduces aldehydes to alcohols), so it can also be assigned to the aldehydes. Furthermore, as suggested by the computation, it is only present when C=C and C=O are *trans* to each other. We also present a new mode (1,400 cm^−1^), which is indicative for C=C―C=O functional groups, hence also coniferyl aldehyde. Due to its low intensity, the fourth band (590 cm^−1^) is less useful but might in fact be the only band unambiguously assigned to coniferyl aldehyde in plant spectra consisting of mixtures of G and S (sinapinaldehyde) units. A comparison of Raman wavenumbers together with assignments is given in Table 1.

**Table 1:** Selected bands of the studied compounds compared with a lignin spectrum obtained from cell corners of spruce (at 532 nm). Assignments of established work as well as this work are given. ^**a**^Coniferyl alcohol which is bound to the lignin structure over the aryl O. This is derived from the spectrum of abietin [Colour table can be viewed at wileyonlinelibrary.com]

Coniferyl alcohol	Abietin	Coniferyl aldehyd	Spruce cell corner	Literature^10^	This work
3,065	3,079		3,075	Aromatic C―H stretch	C―H stretch of ring Φ2
3,000	3,013		3,009	C―H stretch in OCH_3_, asymmetric	C―H stretch of C=C
2,944	2,941		2,941	C―H stretch in OCH_3_, asymmetric	C―H stretch of OCH_3_, asymmetric
2,864	2,876		2,849	C―H stretch in R_3_C‐H	Aliphatic C―H stretch
1,661	1,653	1,670	1,658	Ring conj. C=C stretch of Coniferyl alcohol; C=O stretch of coniferaldehyde	C=C stretch of Coniferyl alcohol, C=O stretch of Coniferyl aldehyde
		1,623	1,620	Ring conj. C=C stretch of coniferaldehyde	C=C stretch of Coniferyl aldehyde
1,607	1,606	1,601	1,598	Aryl ring stretching, symmetric	C=C stretch of ring Φ8b
	1,515	1,516	1,507	Aryl ring stretching, asymmetric	C=C stretch of ring Φ19b
1456	1,455	1,458	1,454	O―CH_3_ deformation; CH_2_ scissoring; guaiacyl ring vibration	CH bending of OCH_3_ and CH_2_
		1,435	1,429	O―CH_3_ deformation; CH_2_ scissoring; guaiacyl ring vibration	CH bending of OCH_3_ and CH_2_; C=C stretch of ring Φ19a
		1,397	1,393	Phenolic O―H bend	C―H bend of Coniferyl aldehyde
	1,324		1,322		C―H bend of ‐O‐4 Coniferyl alcohol^a^
1,298	1,299		1,298	Aryl‐O of aryl‐OH and aryl‐O‐CH_3_; C=C stretch of Coniferyl alcohol	C―H bend of C=C of Coniferyl alcohol
		1,290			C―H bend of C=C of Coniferyl aldehyde
	1,281		1,273	Aryl‐O of aryl‐OH and aryl‐O‐CH_3_; guaiacyl ring (with C=O group) mode	C―H bend of C=C; C=C bend of ring Φ7a
1,222		1,227	1,225	Aryl‐O of aryl‐OH and aryl‐O‐CH_3_; guaiacyl ring (with C=O group) mode	C=C bend of ring Φ13; C―H bend of C=C
1,196	1,197	1,213	1,194	A phenol mode	Aryl‐O‐H bend, CH_3_ rocking
	1,131	1,131	1,139	A mode of coniferaldehyde	C―C stretch of Coniferyl aldehyde
811	804	813	809	Skeletal deformation of aromatic rings, substituent groups, and side chains	C=C stretch of ring Φ1
		584	596	Skeletal deformation of aromatic rings, substituent groups, and side chains	Ring/C=O bend of Coniferyl aldehyde
382			379	Skeletal deformation of aromatic rings, substituent groups, and side chains	C―O―CH_3_ bending?

## 4 CONCLUSIONS

Coniferyl alcohol and aldehyde can be easily identified in the Raman spectrum of lignin, because both compounds are strong Raman scatterers. Most studies attribute this well‐known behavior only to preresonance Raman scattering. Although this is indeed a factor contributing to signal strength, it is not the only one. Often overlooked in studying biological materials is enhancement by charge transfer effects. The latter is independent from the excitation wavelength and enables such molecules to dominate the entire Raman spectrum. It was demonstrated for abietin that a conjugated aromatic moiety is enough to effectively hide the carbohydrate part of the molecule. The spectrum of lignin is another case, where only some substructures account for almost all of the relevant bands. Not all of these molecules have been identified yet, but the implication is two fold.

On the one hand, such substances can be studied despite being present only in low amounts. For example, monolignols and their precursors in the cell wall can be followed using Raman imaging. This selectivity is an advantage over other popular methods like NMR.

On the other hand, this means weak scattering structures can be masked and therefore remain undetected. This seems to be the case in Raman spectra of lignin, where the majority of the aromatic rings (unconjugated ones) can only be seen as weak bands.

IR spectroscopy does not seem to be affected by the aforementioned enhancing effects, so the IR spectrum of lignin incorporates signal from all of its substructures. The downside of this is the severe overlap of bands, which makes it difficult to separate the individual components.

Future work on band assignment has to take this selectivity into account along with intensity tables. In addition to band assignment charts, in Raman spectroscopy, it might be useful to not search for lignin substructures, which effectively have gone silent.

Raman and IR spectroscopy are complimentary analytical tools and depending upon the nature of information desired, one needs to choose the right technique or use those techniques in combination.

## FUNDING INFORMATION

Horizon 2020 Framework Programme, ERC Consolidator grant, Grant Number: 681885. START grant of the Austrian Science Fund, Grant Number: Y‐728‐B16

## Supporting information

Figure S1. Infrared spectra of six different G‐ring models. The aromatic nucleus produces a distinct band complex from 1300‐1100 cm^−1^, which is marked in the figure. By comparing coniferyl alcohol with the other structures, unique bands can be identified and related to the molecule. The additional band at 1395 cm^−1^ might come from the single CH2‐group, although this is unclear. The band at 1084 cm^−1^ is assigned to the C‐C stretch, although from the spectra C‐O stretch is also possible. The O‐H torsion band in coniferyl alcohol is a sign for crystal order.
Figure S2. IR spectra of coniferyl, sinapyl and o‐methoxyaldehyde and alcohol, respectively. Arrows indicate those bands which aldehyde and alcohol share, therefore they do not come from a fermi‐resonance‐splitting.
Figure S3. Uncorrected spectra showing intensity differences of the involved molecules. All measurements were carried out using a 20x (NA 0.4) objective and 0.04 s integration time. Coniferyl aldehyde and alcohol spectra were acquired from crystals, 2‐methoxy‐4‐methylphenol is liquid at room temperature (20 °C). For 532 nm, the laser power was set to 30 mW, for 785 nm to 75 mW. Coniferyl aldehyde always delivers the strongest signal, also far away from resonance conditions. The intensity enhancement is therefore ascribed to other effects (see text). Pre‐resonance Raman effect can therefore not always be held responsible for strong aromatic signal.
Figure S4. Raman spectra (raw) of cinnamaldehyde and cinnamyl alcohol and their respective dehydrogenated forms. The compounds were chosen to represent the base structure of coniferyl alcohol/aldehyde and to remove influence of ring substituents. All of the compounds were measured as liquids at 40°C. Spectra were recorded at 532 nm excitation with 42.3 mW and 0.04 s integration time. The effect of conjugation is seen when comparing the intensity of vibration 8a of phenylpropanol with cinnamyl alcohol. By comparing hydrocinnamaldehyde with cinnamaldehyde, conjugation activates the carbonyl group and intensifies mode 8a. The intensity is further increased by the Я‐effect, a mode which “most strongly couples the geometry to the electron structure”^65^. It is drawn in the inset according to literature^61,65^ and it is seen that all labeled modes coincide with the Я‐mode and are enhanced. The intensity of cinnamyl alcohol and cinnamaldehyde is likely to be enhanced by pre‐resonance as well. Note that the intensity of modes 2 and 12 remains almost constant in all four substances, so that these can be taken as a base reference for comparing the intensity of enhanced modes.
Figure S5. Raman spectra of cinnamaldehyde and cinnamyl alcohol and their respective dehydrogenated forms. All of the compounds were measured as liquids at 40°C. Spectra were recorded at 785 nm excitation with 190 mW and 0.04 s integration time. Pre‐resonance is not expected at this wavelength, so conjugation and Я‐effect can be studied. Note that the ratio of modes 8a and 12 shifted in favor of the latter. Similar to Fig. S4, cinnamyl alcohol shows an enhanced ring mode 8a when compared with phenylpropanol, and this can be solely attributed to the conjugation effect. Conjugation and Я‐effect make cinnamaldehyde the strongest scatterer at this wavelength as well. Because these effects relate to the molecule itself, conjugation and charge‐transfer paths have to be destroyed in order to change this feature.
Figure S6. Infrared spectra of cinnamaldehyde and cinnamyl alcohol and their respective dehydrogenated forms. Except for cinnamyl alcohol, all of the compounds were measured as liquids at 20 °C, so that ATR pressure could not influence their intensity. Spectra were recorded at 32 scans each. Compare the overall intensity of cinnamaldehyde with respect to hydrocinnamaldehyde. Conjugation of the phenyl ring with the carbon double bond is not responsible for the activation of the double bond in the infrared (see arrow), so the effect is attributed to the Я‐effect. Vibrations 19 and 4 have similar intensity in all compounds. It can be deduced from this that enhancement effects play a minor role (single band enhancement, but similar overall intensity) than in Raman (single band and overall intensity enhancement, see Figs. S4 and S5).
Figure S7. Vibrational modes in Wilson^40^/Varsanyi^37^ notation for G‐rings. The 30 modes of benzene are divided into 12 ring carbon modes, 9 hydrogen modes and 9 substituent modes. Arrows depict atomic displacements in the plane of the paper and + and – indicate motion out of the paper plane. The magnitude and direction of displacement have only illustrative character. Calculated displacements of actual molecules can considerably deviate from those shown here, although the principal character of the mode can normally still be recognized.

Supporting info item
