# Supplementary material for: Infrared and Raman spectra of lignin substructures: Coniferyl alcohol, abietin, and coniferyl aldehyde
Source: J Raman Spectrosc. 2019 Apr 1;50(6):778–92. doi: 10.1002/jrs.5588 (PMC6602882; doi:10.1002/jrs.5588)
Supplement: Supplementary file 1 — Figure S1. Infrared spectra of six different G‐ring models. The aromatic nucleus produces a distinct band complex from 1300‐1100 cm−1, which is marked in the figure. By comparing coniferyl alcohol with the other structures, unique bands can be identified and related to the molecule. The additional band at 1395 cm−1 might come from the single CH2‐group, although this is unclear. The band at 1084 cm−1 is assigned to the C‐C stretch, although from the spectra C‐O stretch is also possible. The O‐H torsion band in coniferyl alcohol is a sign for crystal order. Figure S2. IR spectra of coniferyl, sinapyl and o‐methoxyaldehyde and alcohol, respectively. Arrows indicate those bands which aldehyde and alcohol share, therefore they do not come from a fermi‐resonance‐splitting. Figure S3. Uncorrected spectra showing intensity differences of the involved molecules. All measurements were carried out using a 20x (NA 0.4) objective and 0.04 s integration time. Coniferyl aldehyde and alcohol spectra were acquired from crystals, 2‐methoxy‐4‐methylphenol is liquid at room temperature (20 °C). For 532 nm, the laser power was set to 30 mW, for 785 nm to 75 mW. Coniferyl aldehyde always delivers the strongest signal, also far away from resonance conditions. The intensity enhancement is therefore ascribed to other effects (see text). Pre‐resonance Raman effect can therefore not always be held responsible for strong aromatic signal. Figure S4. Raman spectra (raw) of cinnamaldehyde and cinnamyl alcohol and their respective dehydrogenated forms. The compounds were chosen to represent the base structure of coniferyl alcohol/aldehyde and to remove influence of ring substituents. All of the compounds were measured as liquids at 40°C. Spectra were recorded at 532 nm excitation with 42.3 mW and 0.04 s integration time. The effect of conjugation is seen when comparing the intensity of vibration 8a of phenylpropanol with cinnamyl alcohol. By comparing hydrocinnamaldehyde with cinnamaldehyde, conju [file JRS-50-778-s001.zip › JRS_5588_sup-0008-supplementary materials.docx]

Supplementary Material

Compounds dominating lignin: Infrared and Raman spectra of Coniferyl alcohol, Abietin and Coniferyl aldehyde

Peter Bock^1^ and Notburga Gierlinger^1^

^1^Institute of Biophysics, University of Natural Resources and Life Sciences, Vienna, Austria

**CONIFERYL ALCOHOL**

By comparing IR spectra of coniferyl alcohol to similar molecules, there is a single band sticking out at 1084cm^-1^. As of the given molecules (see Fig. S1), only coniferyl alcohol has an additional C-O group; this band would have been assigned to the C-O stretch of the propenol moiety. The DFT calculation contradicts this assignment and shows this vibration to be the C-C stretch of the propenol moiety, regardless whether the hydroxyl group is H-bound or not. Another possibility is coupling of both groups to result in ν_as_ C-C-O and ν_s_ C-C-O. As in either case, the mode is at least localized in the propenol part of the molecule, we follow the DFT result and do not further question it.


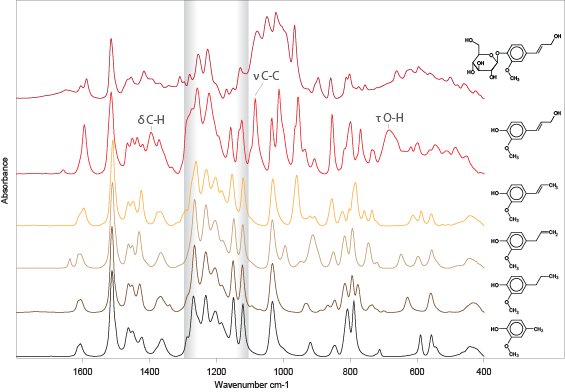


*Figure S1 – Infrared spectra of six different G-ring models. The aromatic nucleus produces a distinct band complex from 1300-1100 cm^-1^, which is marked in the figure. By comparing coniferyl alcohol with the other structures, unique bands can be identified and related to the molecule. The additional band at 1395 cm^-1^ might come from the single CH2-group, although this is unclear. The band at 1084 cm^-1^ is assigned to the C-C stretch, although from the spectra C-O stretch is also possible. The O-H torsion band in coniferyl alcohol is a sign for crystal order.*

**CONIFERYL ALDEHYDE**

**Comments on the 1135cm^-1^ alkenal split**

In the course of studying the vibrational spectra of cinnamaldehydes, we systematically studied the vibrational behavior of the alkenal (C=C-HC=O) group. In doing so, it became apparent that the bands assigned to the C-C stretch are often split. It would be easy to explain this splitting by the occurrence of different species in the sample, i.e. different microenvironments of the respective group.

Crystalline samples complicate the elucidation of this question, as different species can be found. But this normally easy to detect in Raman images, because crystals often have an uneven surface with cracks or defects and the Raman spectra will therefore be different and the extent of crystal splitting can be judged.

For coniferyl aldehyde (split in IR) and p-methoxycinnamaldeyhde (split in Raman) this doublets could be explained with crystal structure (see discussion on this also in the main paper).

4-Acetoxy-3-methoxycinnamaldehyde and sinapin aldehyde also show doublets in the 1130cm^-1^ region, whereas only one carbonyl stretch could be identified (the former molecule of course has two stretches at very different frequencies due to the addition of the acetoxy group, but its C=O stretch can be easily identified by frequency and IR/Raman activity).

Therefore, there remain two options:

First, the second band in question is just by coincidence of similar intensity as and close to the C-C stretch

Or, secondly, the split derives from a Fermi resonance with the C=O bend.

The C=O bend is a bend of medium intensity that appears around 580cm^-1^ and can readily be identified in almost all of the alkenals present. A requirement for fermi resonance is that the two modes of vibration which couple share the same symmetry[^1^](#_ENREF_1). The molecules in question have no (*C_1_*) or very little (*C_s_*) symmetry. This means that *C_s_* already imposes a restriction, because there exist *a’* and *a’’* modes. C-C stretching and C=O bending are both *a’*, so coupling is allowed.

Another requirement, perhaps the more important one, is that the frequency of the overtone has to match the frequency of the fundamental very closely.

A survey of our molecules in question results in:

- Cinnamaldehyde: C-C stretch at 1120cm^-1^, C=O bend at 582cm^-1^; no coupling possible (2x582=1164) and also not observed
- o-Methoxycinnamaldehyde: 1128, 1099, 566 (2x566=1132); coupling possible
- p-Methoxycinnamaldehyde: 1125, 1108, 528 (2x528=1056); no coupling
- Coniferyl aldehyde: 1132, 1116, 579 (2x579=1158); no coupling
- 4-Acetoxy-3-methoxycinnamaldehyde: 1128, 1118, 592/524; C=O unclear, but no coupling in either way
- Sinapinaldehyde: 1139, 1105, 576 (2x576=1152); no coupling
- Furylacrolein: 1125, 591 (2x591=1182); no coupling
- Hexenal: 1152, 1142, 570 (2x570=1140); coupling possible

We therefore reject the hypothesis of fermi resonance in most of the cases (Hexenal, however, might show a fermi resonance, because there are two bands observed, but only one is calculated by the simulation and the frequencies match very well).

This means that a second band unfortunately interferes with the C-C stretch. Luckily, we have also have alcohols of some of the compounds available. Together with the computations we can see, that indeed there is always an explanation for the second band.


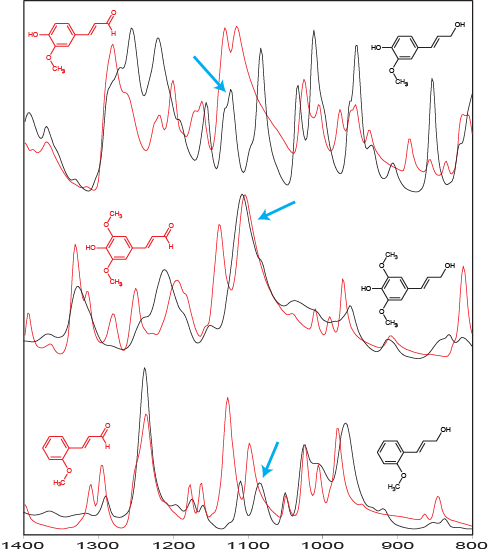


*Figure S2 - IR spectra of coniferyl, sinapyl and o-methoxyaldehyde and alcohol, respectively. Arrows indicate those bands which aldehyde and alcohol share, therefore they do not come from a fermi-resonance-splitting.*

o-Methoxycinnamyl alcohol shows two bands in the same region, the calculation for the aldehyde reveals that the second band is Φ18a.

Sinapyl alcohol shares the second band with the aldehyde; the calculated normal mode is the in-phase-C-O stretch of the methoxy groups.

The band at 1118 in 4-Acetoxy-3-methoxycinnamaldehyde was computed to be a form of C-X ring mode.

Coniferyl alcohol shows a band at 1132 attributed to Φ15. This mode often flanks the C-C stretch in the aldehyde, but especially in Raman the latter is always stronger and thus clearly identified. Based on this, it is apparent that the frequency of the C-C stretch seems to be rather stable by comparing all the compounds in our work, so the 1132 band is assigned as C-C stretch and the 1116 as Φ15 (Φ9b).

**C=C wag and sym-C-O-C stretch**

The assignment of these two bands is difficult, because a survey over our spectra shows that the trans-CH-wag is occurring roughly between 980 and 960 cm^-1^. Since the methoxy group’s symmetric stretch will create a dipole moment change based on its location on the ring, we expect the intensity to vary between different ring substitutions. The trans-CH-wag of the C=C should always create a dipole moment change and its intensity therefore should be invariant to substitution. In p- and o-Methoxycinnamaldehyde, the C-O-C stretch is very weak, whereas in asym-tetrasubstitution like in coniferyl aldehyde or its acetylated analogue, it is of comparable strength to the CH mode. In sinapinaldehyde, both methoxy groups can couple, so the mode is moved out of the range.


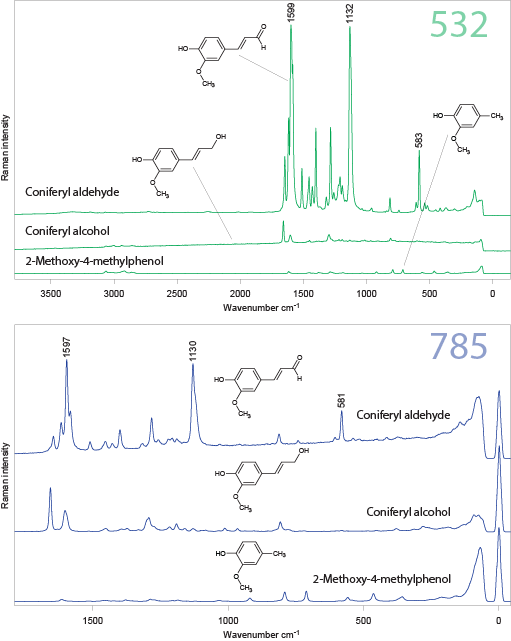


Figure S3 – Uncorrected spectra showing intensity differences of the involved molecules. All measurements were carried out using a 20x (NA 0.4) objective and 0.04 s integration time. Coniferyl aldehyde and alcohol spectra were acquired from crystals, 2-methoxy-4-methylphenol is liquid at room temperature (20°C). For 532 nm, the laser power was set to 30 mW, for 785 nm to 75 mW. Coniferyl aldehyde always delivers the strongest signal, also far away from resonance conditions. The intensity enhancement is therefore ascribed to other effects (see text). Pre-resonance Raman effect can therefore not always be held responsible for strong aromatic signal.


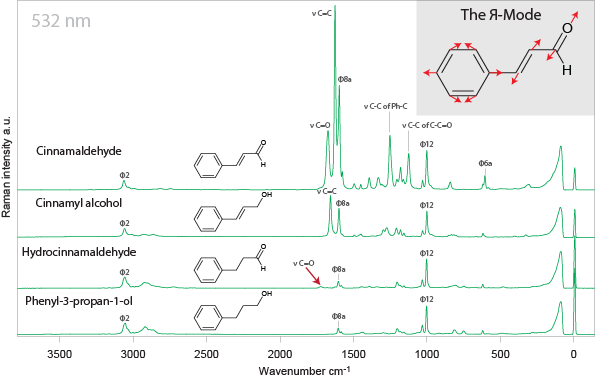


Figure S4 – Raman spectra (raw) of cinnamaldehyde and cinnamyl alcohol and their respective dehydrogenated forms. The compounds were chosen to represent the base structure of coniferyl alcohol/aldehyde and to remove influence of ring substituents. All of the compounds were measured as liquids at 40°C. Spectra were recorded at 532 nm excitation with 42.3 mW and 0.04 s integration time. The effect of conjugation is seen when comparing the intensity of vibration 8a of phenylpropanol with cinnamyl alcohol. By comparing hydrocinnamaldehyde with cinnamaldehyde, conjugation activates the carbonyl group and intensifies mode 8a. The intensity is further increased by the Я-effect, a mode which “most strongly couples the geometry to the electron structure”[^65^](#_ENREF_65). It is drawn in the inset according to literature[^61^](#_ENREF_61)^,^[^65^](#_ENREF_65) and it is seen that all labeled modes coincide with the Я-mode and are enhanced. The intensity of cinnamyl alcohol and cinnamaldehyde is likely to be enhanced by pre-resonance as well. Note that the intensity of modes 2 and 12 remains almost constant in all four substances, so that these can be taken as a base reference for comparing the intensity of enhanced modes.


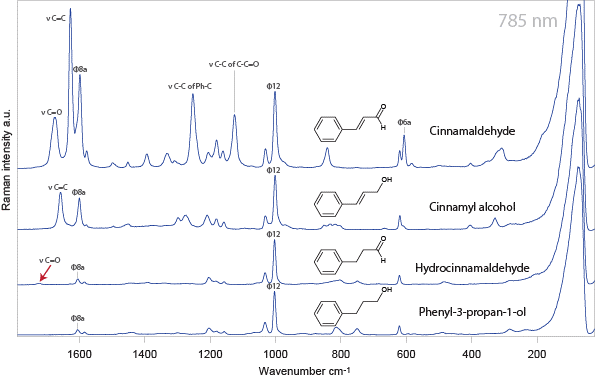


Figure S5 – Raman spectra of cinnamaldehyde and cinnamyl alcohol and their respective dehydrogenated forms. All of the compounds were measured as liquids at 40°C. Spectra were recorded at 785 nm excitation with 190 mW and 0.04 s integration time. Pre-resonance is not expected at this wavelength, so conjugation and Я-effect can be studied. Note that the ratio of modes 8a and 12 shifted in favor of the latter. Similar to Fig. S4, cinnamyl alcohol shows an enhanced ring mode 8a when compared to phenylpropanol, and this can be solely attributed to the conjugation effect. Conjugation and Я-effect make cinnamaldehyde the strongest scatterer at this wavelength as well. Because these effects relate to the molecule itself, conjugation and charge-transfer paths have to be destroyed in order to change this feature.


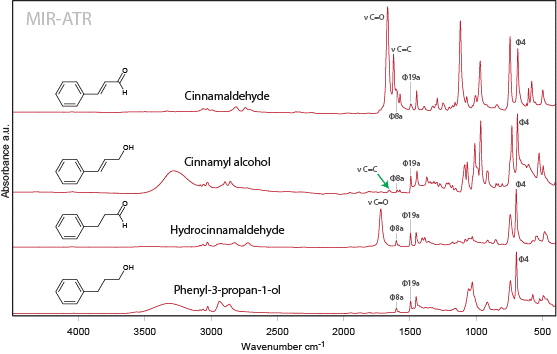


Figure S6 - Infrared spectra of cinnamaldehyde and cinnamyl alcohol and their respective dehydrogenated forms. Exept for cinnamyl alcohol, all of the compounds were measured as liquids at 20°C, so that ATR pressure could not influence their intensity. Spectra were recorded at 32 scans each. Compare the overall intensity of cinnamaldehyde with respect to hydrocinnamaldehyde. Conjugation of the phenyl ring with the carbon double bond is not responsible for the activation of the double bond in the infrared (see arrow), so the effect is attributed to the Я-effect. Vibrations 19 and 4 have similar intensity in all compounds. It can be deduced from this that enhancement effects play a minor role (single band enhancement, but similar overall intensity) than in Raman (single band and overall intensity enhancement, see Figs. S4 and S5).


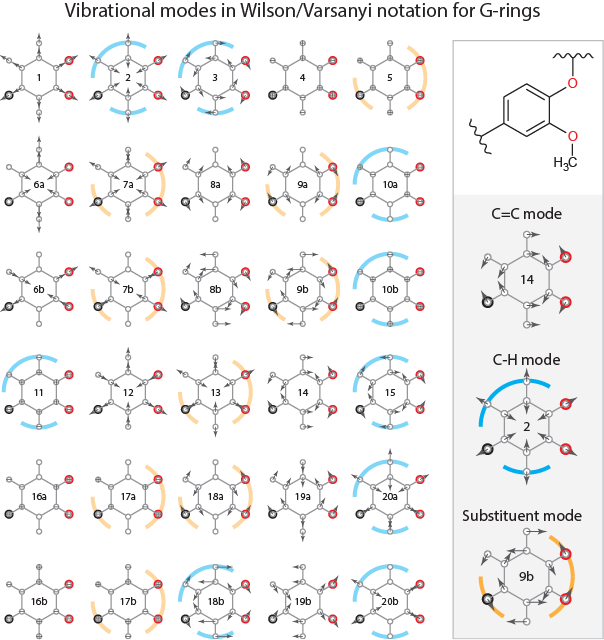


Figure S7 – Vibrational modes in Wilson[^40^](#_ENREF_40)/Varsanyi[^37^](#_ENREF_37) notation for G-rings. The 30 modes of benzene are divided into 12 ring carbon modes, 9 hydrogen modes and 9 substituent modes. Arrows depict atomic displacements in the plane of the paper and + and – indicate motion out of the paper plane. The magnitude and direction of displacement have only illustrative character. Calculated displacements of actual molecules can considerably deviate from those shown here, although the principal character of the mode can normally still be recognized.

(1) Siamwiza MN, Lord RC, Chen MC, Takamatsu T, Harada I, Matsuura H, Shimanouchi T *Biochemistry* **1975**, *14*, 4870.
