# Supplementary material for: Infrared and Raman spectra of lignin substructures: Coniferyl alcohol, abietin, and coniferyl aldehyde
Source: J Raman Spectrosc. 2019 Apr 1;50(6):778–92. doi: 10.1002/jrs.5588 (PMC6602882; doi:10.1002/jrs.5588)
Supplement: Supplementary file 2 — Supporting info item [file JRS-50-778-s002.pdf]

Coniferyl alcohol

erc
European Research Council
Established by the European Commission
Grant No. 681885
www.bionami.at

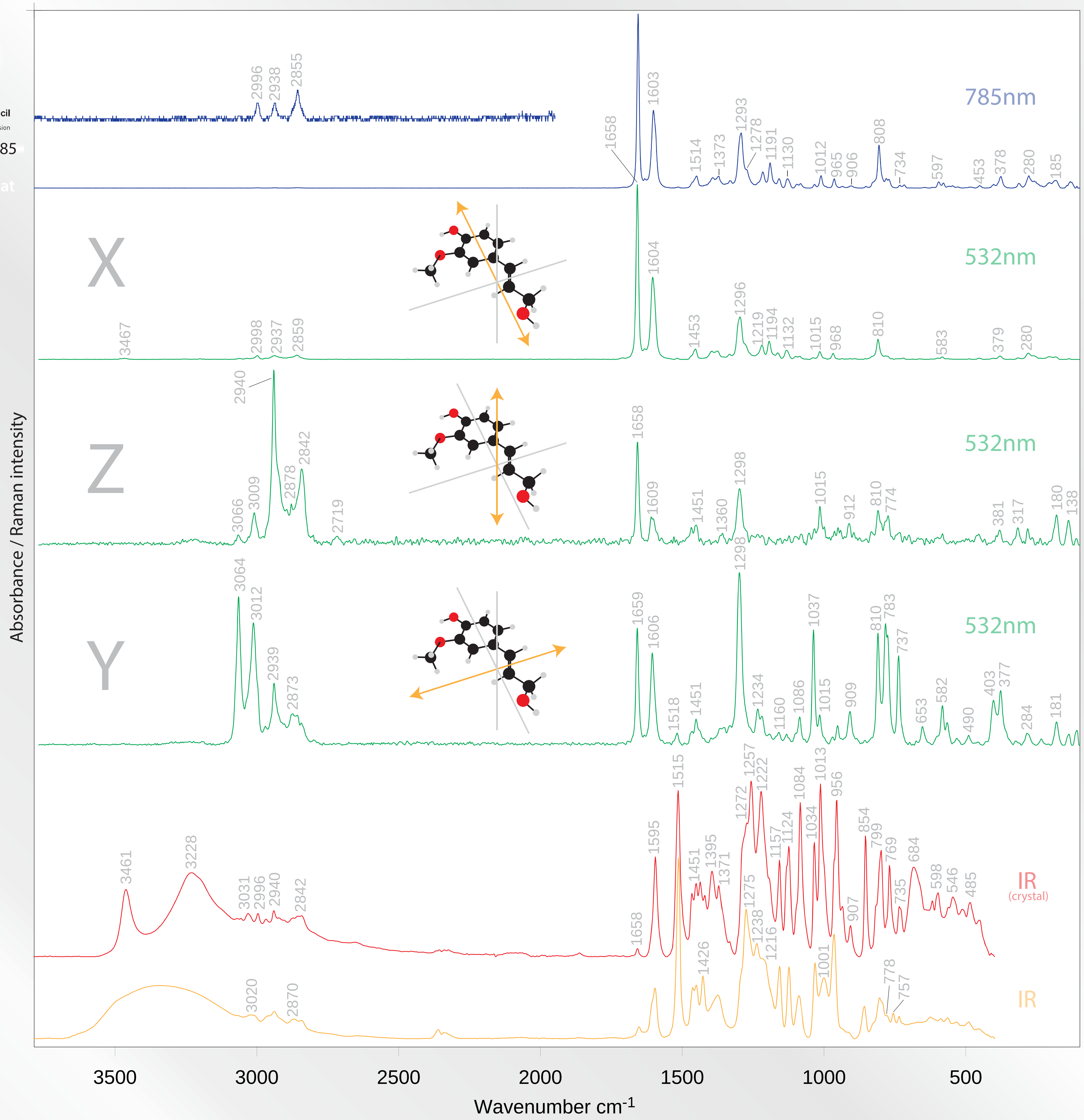

IR		Y	532 X	Z	785 X	
3343	3461		3467			ν O-H of Φ-OH
	3228					ν O-H of OCH_2
	3069	***3064***		3066		ν C-H of ring Φ2
	3056					ν C-H of ring Φ20a
3021	3031					ν C-H of ring Φ20b
		3012		3009		ν C-H of C=C
	2996		2998		2999	ν_{as} C-H of CH_3
2961	2968	2970				ν_{as} C-H of CH_3
2938	2940	2939	2937	***2940***	2941	ν_{as} C-H of CH_3
	2922					ν C-H of CH_3/CH_2
	2906	2903		2895		ν C-H of CH_3/CH_2
2870	2871	2873		2878		ν C-H of CH_3/CH_2
	2855	2857	2859		2858	ν C-H of CH_3/CH_2
2842	2842	2843		***2842***		ν_s C-H of CH_3
		2800				
				2719		$2\delta_{as}$ C-H of CH_3 (overtone)
1653	1658	***1659***	***1658***	***1658***	***1657***	ν C=C
			1633		1631	
1606		***1606***	***1604***	1609	***1604***	ν C=C of ring Φ8b
1596	1595				***1598***	ν C=C of ring Φ8a
1514	***1515***	1518	1518		1516	ν C=C of ring Φ19b
1463	1466	1465			1457	δ_{as} C-H of CH_3; δ_s C-H of CH_2
1451	1451	1451	1453	1451	1450	δ_{as} C-H of CH_3; δ_s C-H of CH_2
	1437					δ C-H
1427	1420	1425				ν C=C of ring Φ19a
	1395	1398	1396		1394	δ C-H
1375	1371	1367	1376		1374	ν C=C of ring Φ14; δ C-H, δ O-H
		1352				δ C-H
	1333	1331	1333		1333	δ C-H of C=C
1293		***1298***	***1296***	***1298***	***1300***	δ C-H of ring Φ3 and C=C
	1285				***1293***	δ C-H of ring Φ3 and C=C
1275	1272		1279		1278	δ C-H of ring Φ3 and C=C
	1257					ν_{ip} [C_Φ-C, C_Φ-O]; δ C=C of ring Φ7a
1238		1234				ν_{op} [C_Φ-C, C_Φ-O]; δ C=C of ring Φ13
1214	***1222***	1219	1219		1216	ν_{op} [C_Φ-C, C_Φ-O]; δ C=C of ring Φ13
1191	1194	1192	1194		1193	δ O-H; γ_r CH_3
					1175	δ O-H; γ_r CH_3
1157	1157	1160	1164		1159	δ C-H of ring Φ18b; γ_r CH_3
1131	1132	1132	1132		1133	δ C-H of ring Φ15
1124	1124	1124				δ C-H of ring Φ15
	1097				1097	
1089	***1084***	1086	1085		1084	ν C-C of CH-CH_2
1032	1034	***1037***			1031	ν C-O of OCH_3
1001	***1013***	1015	1015	1015	1013	ν C-O of CH_2OH
965	964	970	968		966	γ C-H of C=C
	956	952		951		γ C-H of C=C
936	935					γ C-H of ring Φ10a
915	907	909		912	907	ν_s C-O-C; δ C=C of ring Φ7b
858	854					γ C-H of ring and C=C Φ10b
826	816				824	γ C-H of ring and C=C Φ11
805	799	***810***	810	810	***809***	ν C_Φ-OH; ν C=C of ring Φ1 △
797		***783***			784	ν C_Φ-OCH_3; ν C_Φ-C; δ,γ C=C of ring Φ[12,4] ▲
778		***776***		774	775	ν C_Φ-OCH_3; ν C_Φ-C; δ,γ C=C of ring Φ[12,4] ▲
757	769				736	ν C_Φ-OCH_3; ν C_Φ-C; δ,γ C=C of ring Φ[12,4] ▲
736	735	737	721		720	ν C_Φ-OCH_3; ν C_Φ-C; δ,γ C=C of ring Φ[12,4] ▲
	684					τ O-H
		653				τ O-H
625	618					
	598	600			598	
589		582	583		581	γ C_Φ-O; γ C=C of ring Φ16a
565	562	566				δ C=C of ring Φ6a
	546					
533		532				
	513					
490	485	490			488	
456	451		454		456	
		403			404	
		377	379	381	379	
			317	317	315	δ C-O-C
		284	280	282	280	
		234				
			204		208	

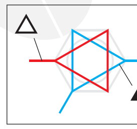

Sample: 223735
Producer: Sigma
Purity: 98%
Mode: Vertex ATR
n_{Acc}: 16/32

λ_{ex}: 532.050 nm
LPO: 30.3 mW
ν (Si): 520.32 cm-1
t_{int}: 0.04371 s
Grating: 600 g/mm

p_{Laser}: 0°/90°
$p_{Spectrometer}$: unpol
Objective: 20x air NA 0.4
measured in: Ethanol
Temperature: 21,2°C

λ_{ex}: 785.008 nm
LPO: 78 mW
ν (Si): 519.93 cm-1
t_{int}: 5.09750 s
Grating: 1200 g/mm

p_{Laser}: 0°
$p_{Spectrometer}$: unpol
Objective: 20x air NA 0.4
measured in: crystalline
Temperature: 20,8°C

ν...stretch, δ...in-plane bend,
γ...out-of-plane bend, τ...torsion,
γ_r...rocking, γ_w...wagging, γ_t...twisting
Φ...ring (Varsanyi), d...degenerate
s...symmetric/scissoring, as... a(nti-)symmetric,
ip...in-phase, op...out-of-phase

Peter Bock
12.11.2018

Bock and Gierlinger, J Raman Spectrosc. 2019; 1-15. https://doi.org/10.1002/jrs.5588

Abietin (Coniferin)

European Research Council
Established by the European Commission
Grant No. 681885
www.bionami.at

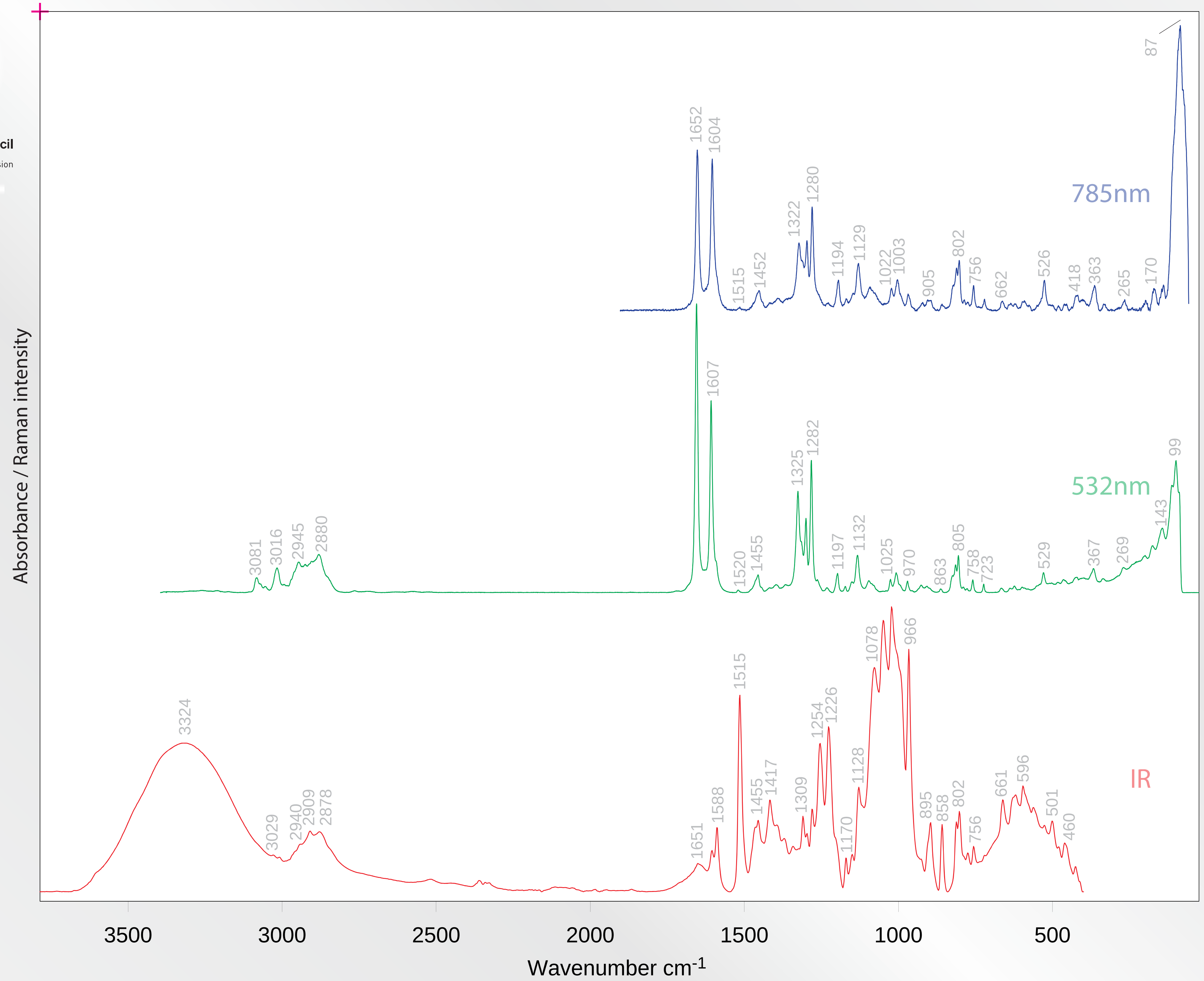

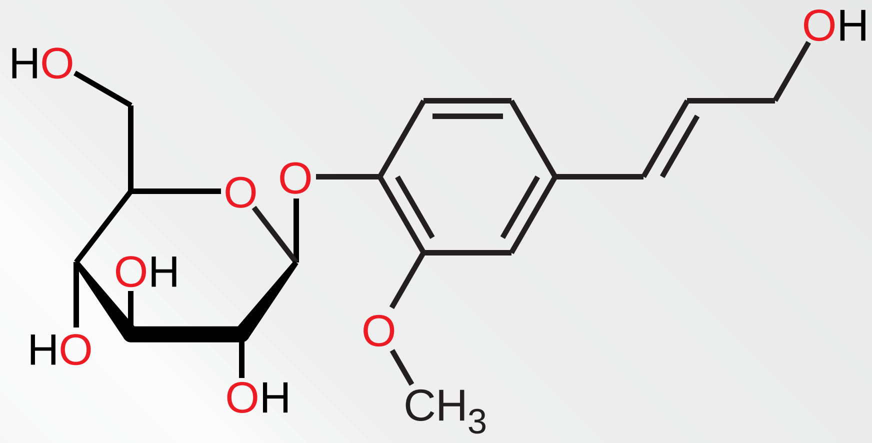

Sample:	SMB00103	Mode:	Vertex ATR
Producer:	Sigma	n_{Acc}:	32
Purity:	90%		
State:	crystalline		
Temperature:	23°C		
measured in:	H_2O		
λ_{ex}:	532.001 nm	λ_{ex}:	785.008 nm
LPO:	30.2 mW	LPO:	220.0 mW
ν (Si):	518.68 cm^{-1}	ν (Si):	520.23 cm^{-1}
t_{int}:	5.09756 s	t_{int}:	5.09756 s
Grating:	1800 g/mm	Grating:	1200 g/mm
P_{Laser}:	0°	P_{Laser}:	0°
$P_{Spectrometer}$:	unpol	$P_{Spectrometer}$:	unpol
Objective:	20x air NA 0.4	Objective:	20x air NA 0.4

IR	532	785	
3324			ν O-H
3312			ν O-H
	3256		ν O-H
	3210		ν O-H
	3081		ν C-H of ring Φ2
	3053		ν C-H of ring Φ20a
3029			ν C-H of ring Φ20b
	3016		ν C-H of C=C
3009			ν C-H of sp^3-carbon
	2993		ν C-H of sp^3-carbon
2940	2945		ν C-H of sp^3-carbon
	2924		ν C-H of sp^3-carbon
2909	2904		ν C-H of sp^3-carbon
2878	2880		ν C-H of sp^3-carbon
	2764		
1651	***1654***	***1652***	ν C=C
1605	***1607***	***1604***	ν C=C of ring Φ8b
1588			ν C=C of ring Φ8a
1515	1520	1515	ν C=C of ring Φ19b
1464			δ C-H
1455	1455	1452	δ C-H
1417			ν C=C of ring Φ19b
1394	1397	1391	δ C-H
1370	1366		
1342			
1324	***1325***	***1322***	ν C=C of ring Φ14; δ C-H of C=C
1309			δ C-H of ring Φ3; δ C-H of C=C
1296	***1299***	***1297***	δ C-H of ring Φ3; δ C-H of C=C
1280	***1282***	***1280***	δ C-H of ring Φ3; δ C-H of C=C
1254	1262		ν_{ip} [C_Φ-C, C_Φ-O]; δ C=C of ring Φ7a; δ C-H
1226	1232	1228	ν_{op} [C_Φ-C, C_Φ-O]; δ C=C of ring Φ13; δ C-H
1202	1197	1194	γ_r CH_3; δ O-H
1170	1172	1170	γ_r CH_3; δ O-H
1150	1150		δ C-H of ring Φ18b
1128	1132	1129	δ C-H of ring Φ15; γ_r CH_3; γ_t CH_2
	1096	1091	ν [C-C, C-O] of glucose
1078			ν C-C of CH_2OH of coniferyl alcohol moiety
1049			ν [C-C, C-O] of glucose
1022	1025	1022	ν [C-C, C-O] of glucose
1005	1007	1003	ν C-O of CH_2OH of coniferyl alcohol moiety
994			ν [C-C, C-O] of glucose
966	970	969	γ_w C-H of C=C
926	926	921	γ C-H of ring Φ10a
904	907	905	ν_s C-O-C; δ C=C of ring Φ7b
895			
858	863	858	γ C-H of ring Φ10b
	824	822	
812	***814***	***811***	γ_t C-H of C=C and ring Φ11
802	***805***	***802***	ν C_Φ-OH; ν C=C of ring Φ1 △
	788	786	
775	777	775	
756	758	756	ν C_Φ-OCH_3; ν C_Φ-C; δ,γ C=C of ring Φ[12,4] ▲
720	723	720	ν C_Φ-OCH_3; ν C_Φ-C; δ,γ C=C of ring Φ[12,4] ▲
661	665	662	
	637	635	
620	623	622	
596	598	591	
562			
526	529	526	
501	502		
480	482	481	
460	463	454	
425	422	418	
	397	403	
	367	363	
	336	334	
	269	265	
	230		
		217	
	199	198	
	175	170	
	143	145	
	112		
	99		
	88	87	
		85	

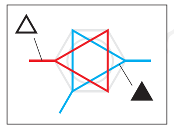

ν...stretch, δ...in-plane bend, γ...out-of-plane bend, τ...torsion, γ_r...rocking, γ_w...wagging, γ_t...twisting Φ...ring (Varsanyi), d...degenerate s...symmetric/scissoring, as... a(nti-)symmetric, ip...in-phase, op...out-of-phase

Peter Bock
04.06.2018

Bock and Gierlinger, J Raman Spectrosc. 2019; 1-15. https://doi.org/10.1002/jrs.5588

European Research Council
Established by the European Commission
Grant No. 681885
www.bionami.at

Coniferyl aldehyde

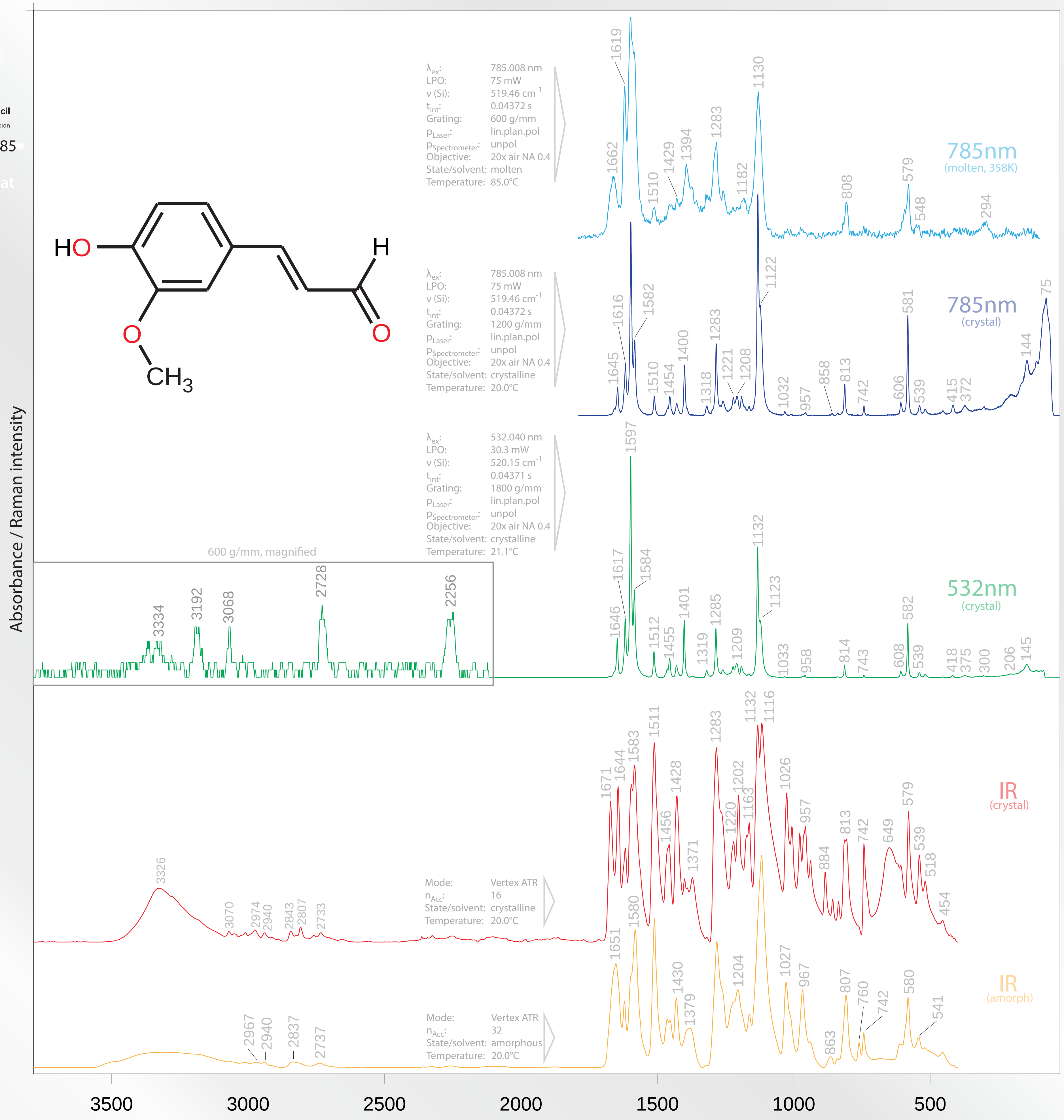

Sample: 223735
Producer: Sigma
Purity: 98%

785		532	IR		
		3334			ν O-H
			3326	3281	ν O-H
		3192			2ν C=C of ring Φ8b (2 x 1597)
		3068	3070		ν C-H of ring
			3049		ν C-H of ring
			3009	3009	ν C-H of ring and C=C
			2974	2967	ν_{as} C-H of CH_3
			2940	2940	ν_{as} C-H of CH_3
			2843	2837	ν C-H of CH_3
			2807		ν C-H of HC=O
			2759		ν C-H of HC=O
		2728	2733	2737	ν C-H of HC=O; combination band (1597 + 1132)
		2256			2ν C-C of C-C=O (2 x 1132)
1662	1657		1671		ν C=O
	1645	1646	1644	1651	ν C=O (H-bonded)
1619	1616	1617	1617	1620	ν C=C
1598	***1596***	***1597***	1595	1595	ν C=C of ring Φ8b
	1582	1584	1583	***1580***	ν C=C of ring Φ8a
1510	1510	1512	***1511***	***1509***	ν C=C of ring Φ19b
	1461	1463		1463	δ_{as} C-H of CH_3
1456	1454	1455	1456	1452	δ_{as} C-H of CH_3
1429	1427	1429	1428	1430	ν C=C of ring Φ19a
1394	1400	1401	1400		δ C-H of HC=O and C=C
			1390		δ C-H of HC=O and C=C
1375			1371	1379	ν C=C of ring Φ14; δ O-H
1356					
	1318	1319	1318	1319	δ C-H of sp^2-C; Φ3
1283	1283	1285	***1283***	***1281***	δ C=C of ring Φ7a; δ C-H of sp^2-C; ν_{ip} [C_Φ-O,C_Φ-C]
1259	1259	1259	1264	1262	δ C=C of ring Φ7a; δ C-H of sp^2-C; ν_{ip} [C_Φ-O,C_Φ-C]
	1221	1222	1220	1221	δ C=C of ring Φ13; ν C_Φ-O, C_Φ-C; δ C-H of C=C
	1208	1209	1202	1204	δ O-H; γ_r C-H of CH_3
	1191	1192			δ O-H; γ_r C-H of CH_3
1182			1172		δ C-H of ring Φ18b; γ_r C-H of CH_3
	1162	1166	1163	1162	δ C-H of ring Φ18b; γ_r C-H of CH_3
1130	***1131***	***1132***	***1132***		ν C-C of C-C=O
	1122	1123	***1116***	***1117***	δ C-H of ring Φ15; ν C-C of C-C=O
1022	1032	1033	1026	1027	ν C-O of O-CH_3; γ_r C-H of CH_3
			1006	1012	γ_{ip} [C-H of C=C-C=O]
975			978	967	γ_{op} [C-H of C=C-C=O]
	957	958	957		ν_s [C-O-C]; δ C=C of ring Φ7b
			938	940	γ C-H of ring Φ10a
			884		γ_{op} [C-H of C=C-C=O] and ring (lone H)] Φ10b
	858		857	863	γ_{op} [C-H of C=C-C=O] and ring (lone H)] Φ10b
	838	839	835	838	γ C-H of ring Φ11
	813	814	813		ν C=C of ring Φ1; ν C_Φ-OH △
808			806	807	ν C=C of ring Φ1; ν C_Φ-OH △
			762	760	γ C=C of ring Φ4
	742	743	742	742	δ C=C of ring Φ12; ν_{ip} [C_Φ-OCH_3, C_Φ-C] ▲
	728		731		δ C=C of ring Φ12; ν_{ip} [C_Φ-OCH_3, C_Φ-C] ▲
			650		τ O-H
			620		
	606	608	608	611	
579	***581***	***582***	***579***	580	δ C=C of ring Φ6a; δ C=O
548	539	539	539	541	
	518	519	518	519	
	452		454	454	
	415	418	425	415	
	372	375			
294	302	300			
	202	206			
	144	145			
	110				
	75				

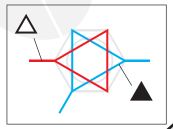

ν...stretch, δ...in-plane bend, γ...out-of-plane bend, τ...torsion, γ_r...rocking, γ_w...wagging, γ_t...twisting
Φ...ring (Varsanyi), d...degenerate
s...symmetric/scissoring, as... a(nti-)symmetric, ip...in-phase, op...out-of-phase

Peter Bock
16.08.2018

v2.1

Bock and Gierlinger, J Raman Spectrosc. 2019; 1-15. https://doi.org/10.1002/jrs.5588

Vanillylidenacetone

European Research Council
Established by the European Commission
Grant No. 681885
www.bionami.at

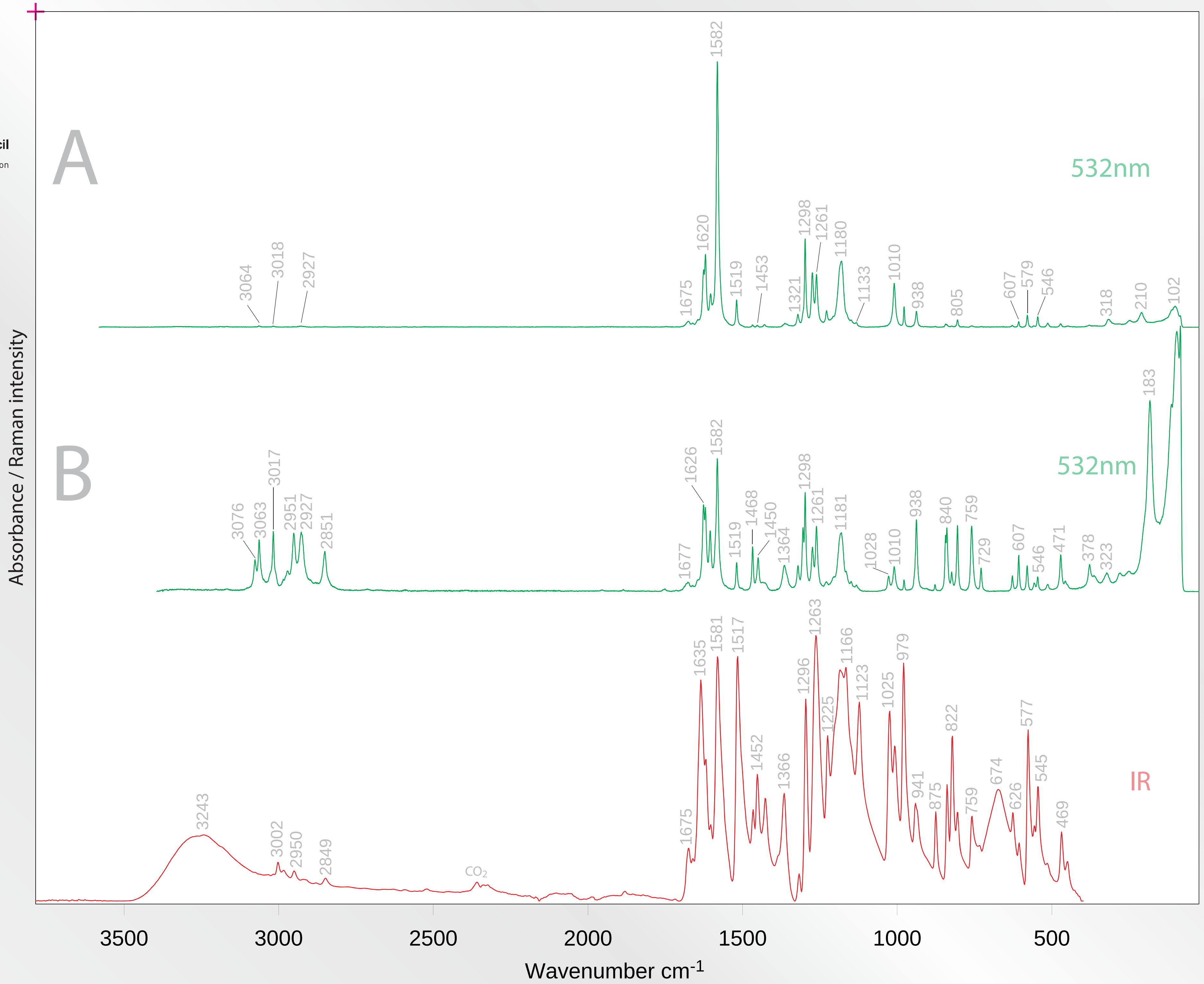

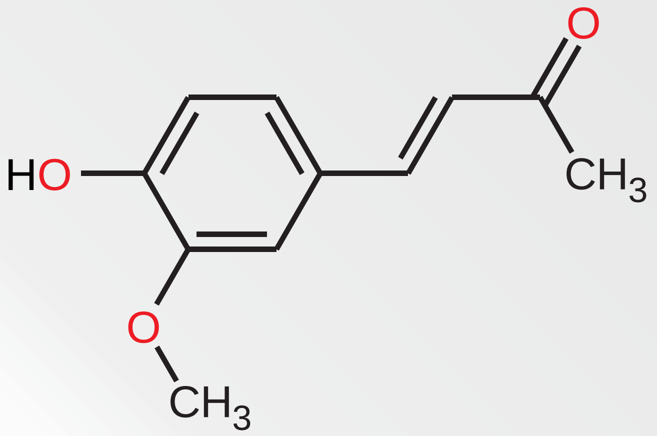

Sample: 90609
Producer: Sigma
Purity: 98%
State: crystalline
Temperature: 2°C
measured in: neat

Mode: Vertex ATR
n_{Acc}: 32

λ_{ex}: 532.050 nm
LPO: 5.00 mW
ν (Si): 520.29 cm^{-1}
t_{int}: 1-5 s
Grating: 1800 g/mm
P_{Laser}: 0°
$P_{Spectrometer}$: unpol
Objective: 20x air NA 0.4

IR	532 A	532 B
3243		
3002		3076
	3064	3063
	3018	3017
2984		
		2971
2950		2951
2920	2927	2927
2879		
2849		2851
1675	1675	1677
1661	1662	
1635	1645	
	1626	1626
1619	1620	1620
1603	1603	1605
1581	***1582***	***1582***
1517	1519	1519
1466	1468	1468
1452	1453	1450
1426	1428	
1366	1363	1364
1317	1321	1321
		1305
1296	***1298***	***1298***
	1274	1274
1263	1261	1261
1225	1229	1229
1185	***1180***	1181
1166	1150	1149
1123	1133	1132
1025		1028
1008	1010	1010
979	978	978
941	938	938
875	878	878
839	843	844
		840
822	825	824
805	805	805
759	758	759
734	728	729
674		
626	628	628
606	607	607
577	579	580
557		557
545	546	546
515	513	514
469	472	471
450	448	456
	379	378
		364
	318	323
		279
	248	251
	210	***183***
	102	***113***
		96
	85	85

Peter Bock
31.07.2018

ν...stretch, δ...in-plane bend, γ...out-of-plane bend, τ...torsion, γ_r...rocking, γ_w...wagging, γ_t...twisting Φ...ring (Varsanyi), d...degenerate
s...symmetric/scissoring, as... a(nti-)symmetric, ip...in-phase, op...out-of-phase

Bock and Gierlinger, J Raman Spectrosc. 2019; 1-15. https://doi.org/10.1002/jrs.5588

European Research Council
Established by the European Commission
Grant No. 681885
www.bionami.at

trans-3-(2-Furyl)acrolein

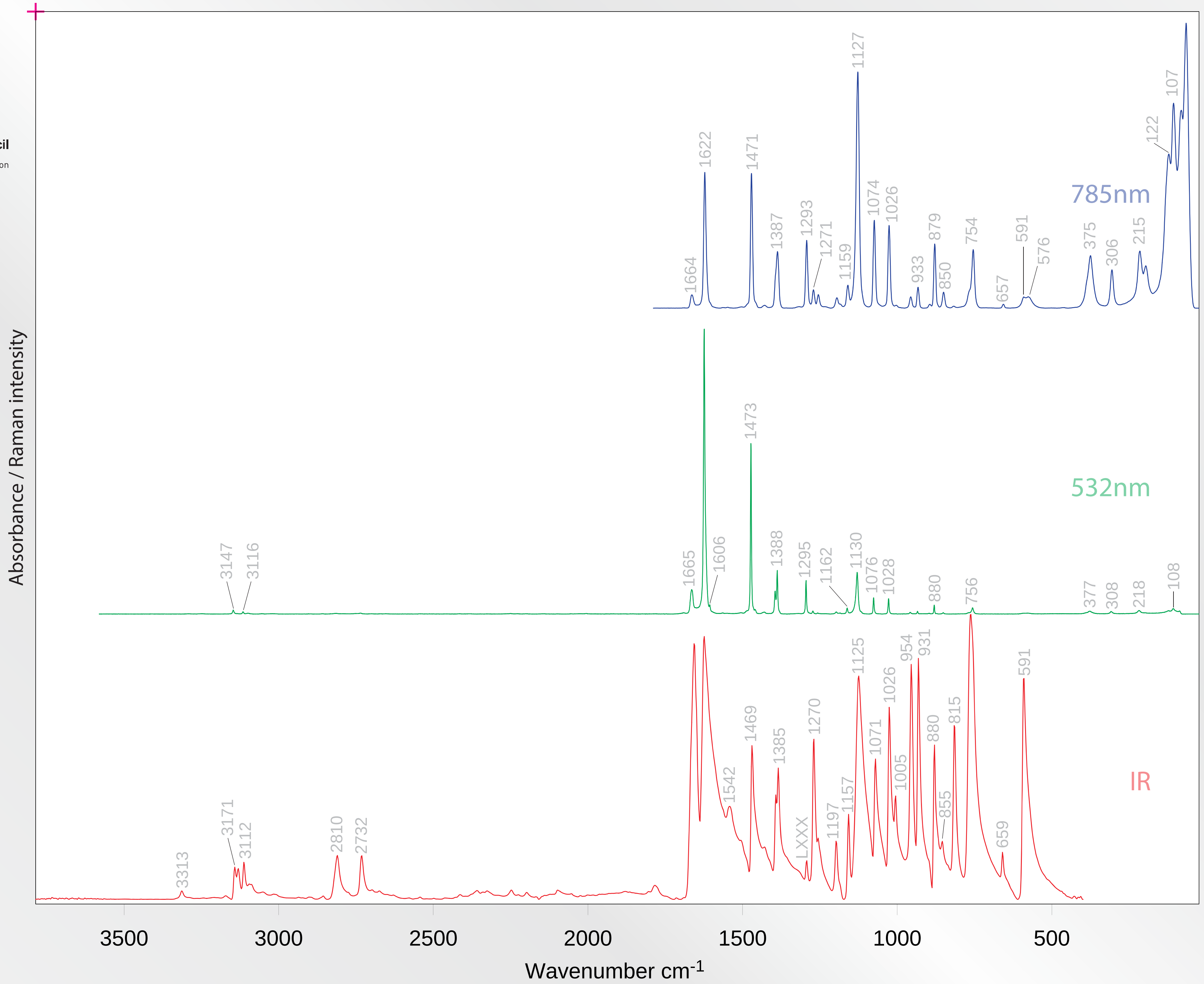

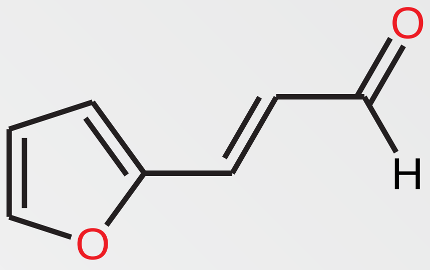

Sample:	W249408	Mode:	Vertex ATR
Producer:	Sigma	n_{Acc}:	32
Purity:	99%		
State:	crystalline		
Temperature:	23°C		
measured in:	neat		

λ_{ex}:	532.001 nm	λ_{ex}:	785.008 nm
LPO:	37.5 mW	LPO:	124.6 mW
ν (Si):	521.32 cm^{-1}	ν (Si):	520.65 cm^{-1}
t_{int}:	0.19756 s	t_{int}:	1.0975 s
Grating:	1800 g/mm	Grating:	600 g/mm
p_{Laser}:	0°	p_{Laser}:	0°
$p_{Spectrometer}$:	unpol	$p_{Spectrometer}$:	unpol
Objective:	20x air NA 0.4	Objective:	20x air NA 0.4

IR	532	785
3313		
3142	3147	
3131		
3112	3116	
3095		
2810	2816	
2732	2735	
1656	1665	1664
1624	***1624***	***1622***
	1606	
1542		
1469	***1473***	***1471***
	1460	
1429	1430	1429
1393	1395	
1385	1388	1387
1293	1295	1293
1270	1273	1271
1256	1257	1255
1197	1197	1195
1157	1162	1159
1125	1130	***1127***
1071	1076	1074
1026	1028	1026
1005		1003
954	958	956
931	934	933
		895
880	880	879
855	851	850
815		817
763	756	754
659		657
591		591
	578	576
	377	375
	308	306
	218	215
		196
		122
	108	***107***
	87	***82***
		66

Peter Bock
31.07.2018

ν...stretch, δ...in-plane bend, γ...out-of-plane bend, τ...torsion, γ_r...rocking, γ_w...wagging, γ_t...twisting Φ...ring (Varsanyi), d...degenerate
s...symmetric/scissoring, as... a(nti-)symmetric, ip...in-phase, op...out-of-phase

Bock and Gierlinger, J Raman Spectrosc. 2019; 1-15. https://doi.org/10.1002/jrs.5588

trans-2-Hexen-1-al

European Research Council
Established by the European Commission
Grant No. 681885
www.bionami.at

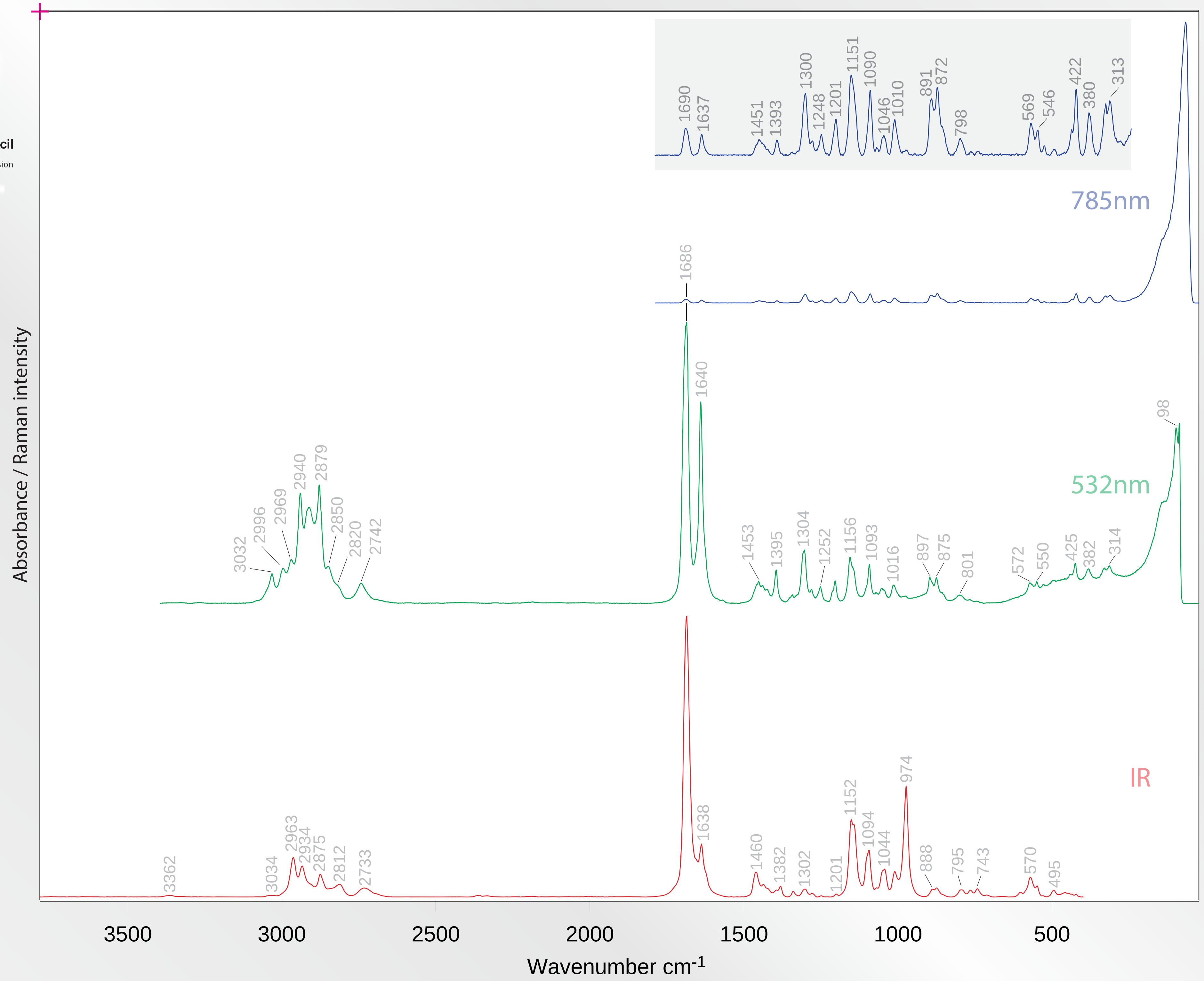

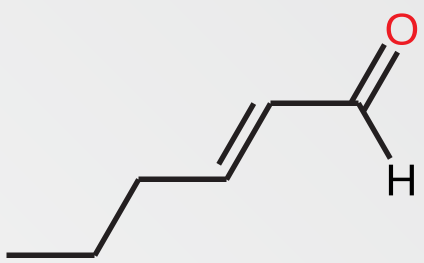

Sample: 132659
Producer: Sigma
Purity: 99%
State: liquid
Temperature: 23°C
measured in: -

Mode: Vertex ATR
n_{Acc}: 32

	532 nm	785 nm
λ_{ex}:	532.001 nm	785.008 nm
LPO:	30.2 mW	114.4 mW
ν (Si):	521.32 cm^{-1}	520.42 cm^{-1}
t_{int}:	5.0975 s	1.0975 s
Grating:	1800 g/mm	600 g/mm
p_{Laser}:	0°	0°
$p_{Spectrometer}$:	unpol	unpol
Objective:	20x air NA 0.4	20x air NA 0.4

IR	532	785
3362		
3034	3032	
	2996	
2963	2969	
2934	***2940***	
	2911	
2875	***2879***	
	2850	
2812	2820	
2733	2742	
1686	***1686***	1690
1638	***1640***	1637
1460	1453	1451
1437	1439	
	1426	
1395	1395	1393
1382		
1340	1343	
1302	1304	1300
1279	1281	1281
1249	1252	1248
1201	1204	1201
1152	1156	***1151***
1143		
1094	1093	1090
	1072	1068
1044	1052	1046
1011	1016	1010
974	974	
888	897	891
875	875	***872***
795	801	798
765		
743	746	
603		
570	572	569
549	550	546
	529	525
495	495	495
458		
	442	438
422	425	422
	382	380
	330	326
	314	313
	137	143
	98	

Peter Bock
31.07.2018

ν...stretch, δ...in-plane bend, γ...out-of-plane bend, τ...torsion, γ_r...rocking, γ_w...wagging, γ_t...twisting Φ...ring (Varsanyi), d...degenerate
s...symmetric/scissoring, as... a(nti-)symmetric, ip...in-phase, op...out-of-phase

Bock and Gierlinger, J Raman Spectrosc. 2019; 1-15. https://doi.org/10.1002/jrs.5588

European Research Council
Established by the European Commission
Grant No. 681885
www.bionami.at

(1R)-(-)-Myrtenal

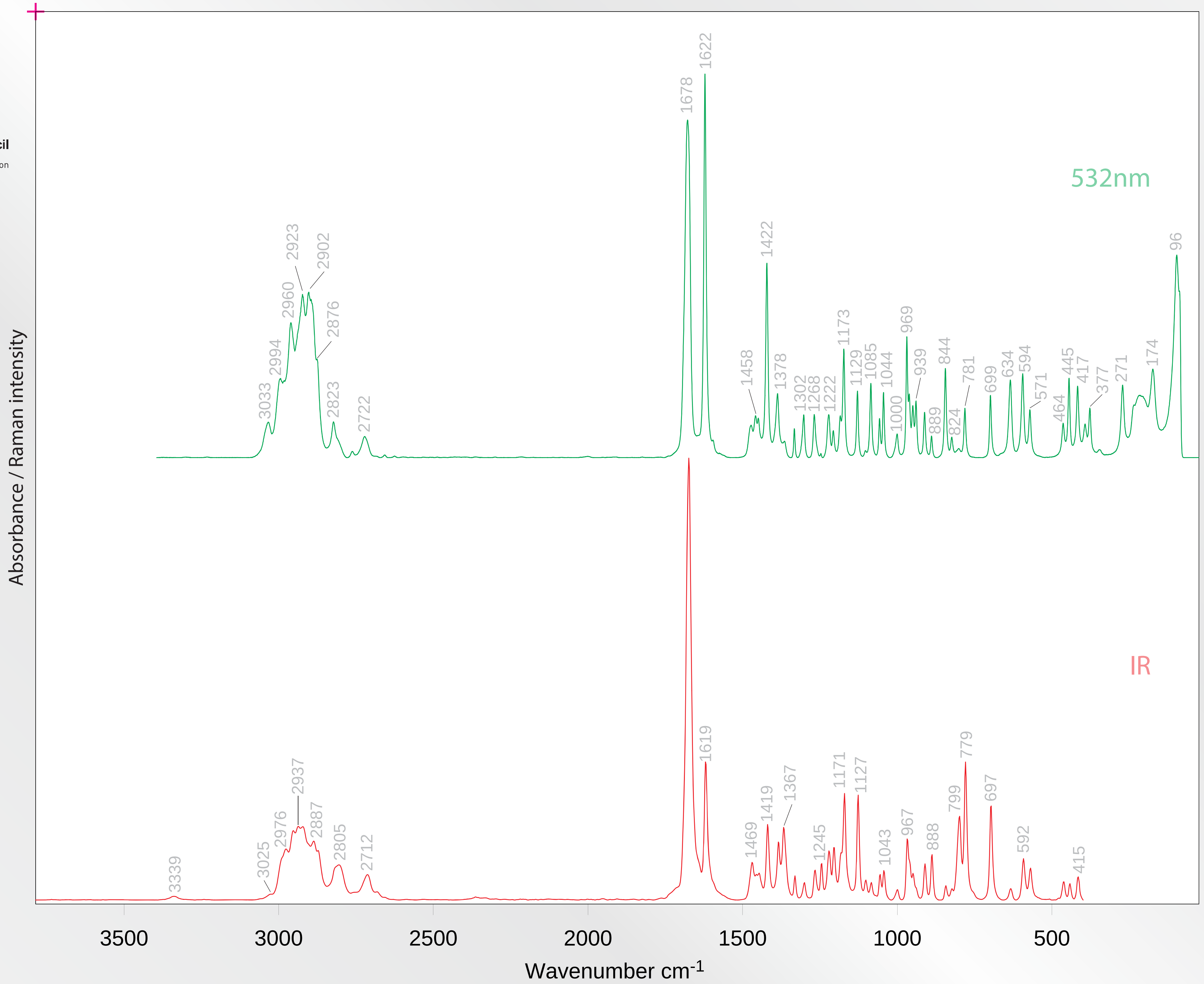

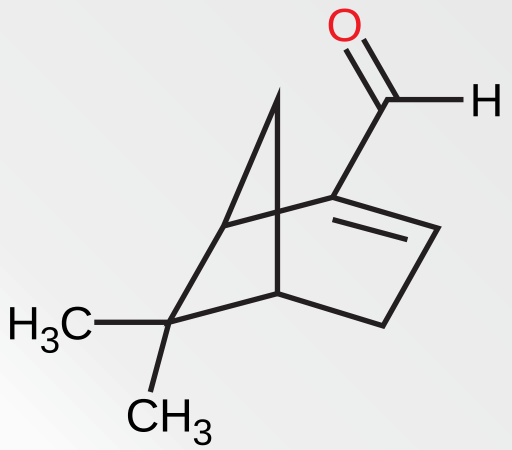

Sample:	W339504
Producer:	Sigma
Purity:	97%
State:	liquid
Temperature:	20.8°C
measured in:	neat

Mode:	Vertex ATR
n_{Acc}:	32

λ_{ex}:	532.001 nm
LPO:	30.0 mW
ν (Si):	-
t_{int}:	5.09756 s
Grating:	1800 g/mm
p_{Laser}:	0°
$p_{Spectrometer}$:	unpol
Objective:	20x air NA 0.4

IR	532
3339	
3025	3033
	2994
2976	
	2960
2953	
2937	
2922	***2923***
	2902
2887	
2872	
2805	2823
	2762
2712	2722
1674	***1678***
1619	***1622***
1469	1473
1455	1458
1447	1449
1419	***1422***
1384	1387
1367	1365
1331	1333
1300	1302
1266	1268
1245	1247
1220	1222
1204	1207
1182	1184
1171	1173
1127	1129
1102	1103
1084	1085
1056	1057
1043	1044
999	1000
967	969
	961
949	950
	939
910	912
888	889
843	844
823	824
799	801
779	781
697	699
633	634
592	594
569	571
462	464
442	445
415	417
	393
	377
	347
	271
	215
	174
	96

Peter Bock
31.07.2018

ν...stretch, δ...in-plane bend, γ...out-of-plane bend, τ...torsion, γ_r...rocking, γ_w...wagging, γ_t...twisting Φ...ring (Varsanyi), d...degenerate
s...symmetric/scissoring, as... a(nti-)symmetric, ip...in-phase, op...out-of-phase

Bock and Gierlinger, J Raman Spectrosc. 2019; 1-15. https://doi.org/10.1002/jrs.5588

2-Methoxy-4-methylphenol

erc
European Research Council
Established by the European Commission
Grant No. 681885
www.bionami.at

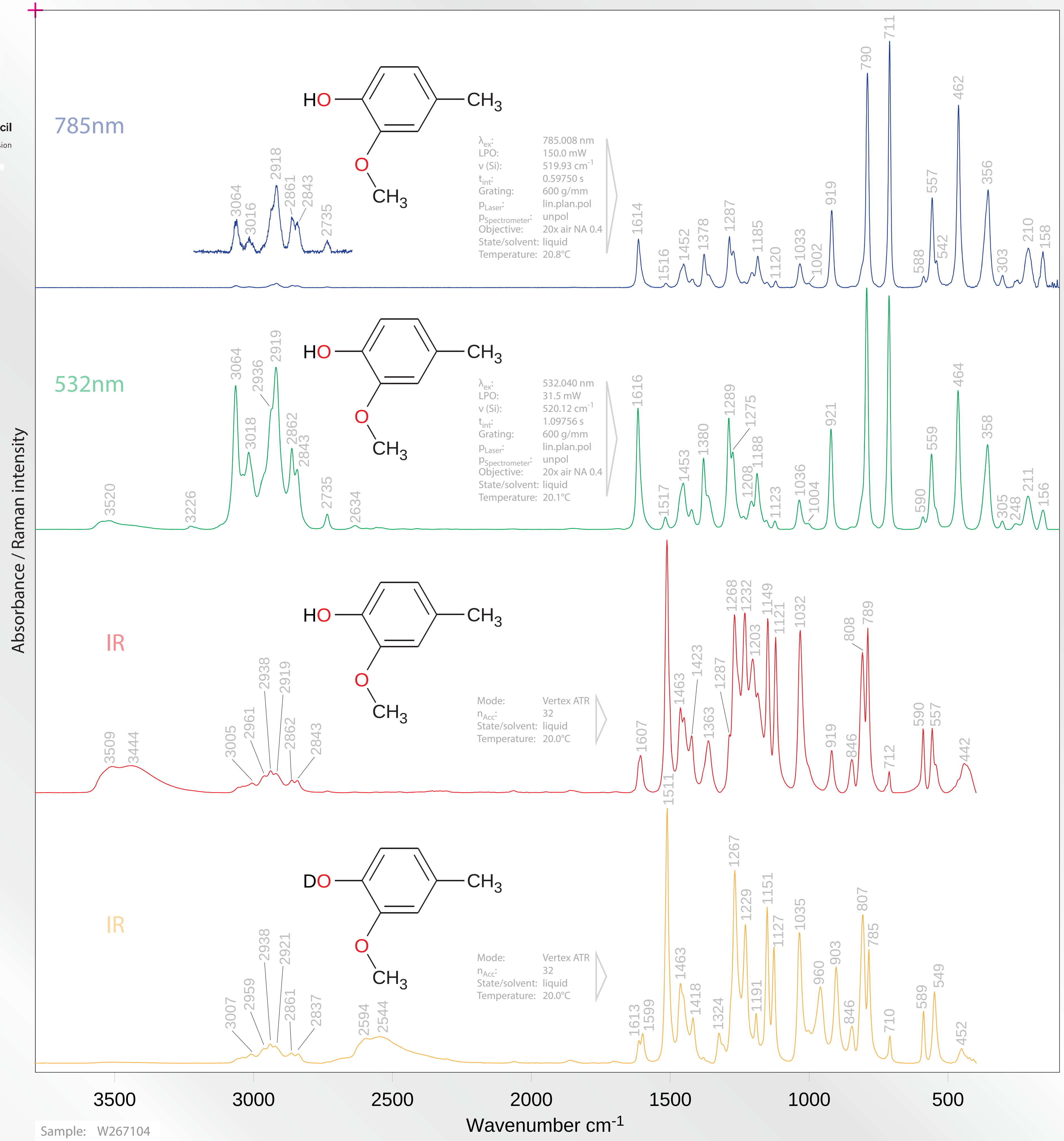

Sample: W267104
Producer: Sigma
Purity: 98%

785	532	IR		
	3520	3509		ν O-H
	3226	3444		ν O-H
3064	***3064***	3056	3053	ν C-H of ring Φ2
	3038	3038	3037	
3016	3018			
		3005	3007	
		2961	2959	ν_{as} C-H of CH_3
2936	2936	2938	2938	ν_{as} C-H of CH_3
2919	***2919***	2919	2921	ν_s C-H of CH_3
2861	2862	2862	2861	ν_s C-H of CH_3
2843	2843	2843	2837	ν_s C-H of CH_3
2735	2735			$2\delta_{as}$ C-H of CH_3 (overtone)
	2634			
			2594	ν O-D
			2544	ν O-D
1614	1616		1613	ν C=C of ring Φ8b
		1607	1599	ν C=C of ring Φ8a
1516	1517	***1512***	1511	ν C=C of ring Φ19b
		1463	1463	
1452	1453	1450	1454	
1422	1423	1423		ν C=C of ring Φ19a
1378	1380	1380	1380	ν C=C of ring Φ14-3; δ O-H △
1360	1364	1363		ν C=C of ring Φ14-3; δ O-H △
			1324	ν C=C of ring Φ14-3; δ O-D △
			1310	ν C=C of ring Φ14-3; δ O-D △
1287	1289	1287	1280	ν C=C of ring Φ14+3; δ C-H ▲
1273	1275	***1268***	1267	ν_{ip} C_Φ-X; δ C=C of ring Φ7a
1235	1236	***1232***	1229	ν_{op} C_Φ-X; δ C=C of ring Φ13
1206	1208	1203		δ O-H
1185	1188	1185	1191	γ_r C-H of CH_3
1152	1155	***1149***	1151	δ C-H of ring Φ18b
1120	1123	1121	1127	δ C-H of ring Φ15
1033	1036	1032	1035	ν C-O of O-CH_3
1001		1002	1003	γ_r C-H of ring Φ10a
			960	δ O-D
919	921	919	903	ν_s C-O-C; δ C=C of ring Φ7b
	849	846	846	γ C-H of ring Φ10b
		808	807	γ C-H of ring Φ11
790	***793***	***789***	785	ν C=C of ring Φ1△
		723	723	γ C=C of ring Φ4
711	***712***	712	710	δ C=C of ring Φ12▲
588	590	590	589	γ C=C of ring Φ16a
557	559	557		δ_{op} C_Φ-X; δ C=C of ring Φ9b
542	542	545	549	δ_{op} C_Φ-X; δ C=C of ring Φ9b
462	464		452	δ C=C of ring Φ6a
		442		
			424	
			410	
356	358			δ_{op} C_Φ-X; δ C=C of ring Φ9a
303	305			
250	253			
210	211			
158	156			

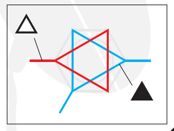

Peter Bock
14.12.2018

ν...stretch, δ...in-plane bend, γ...out-of-plane bend, τ...torsion, γ_r...rocking, γ_w...wagging, γ_t...twisting Φ...ring (Varsanyi), d...degenerate
s...symmetric/scissoring, as... a(nti-)symmetric, ip...in-phase, op...out-of-phase

Bock and Gierlinger, J Raman Spectrosc. 2019; 1-15. https://doi.org/10.1002/jrs.5588

European Research Council
Established by the European Commission

Isoeugenol

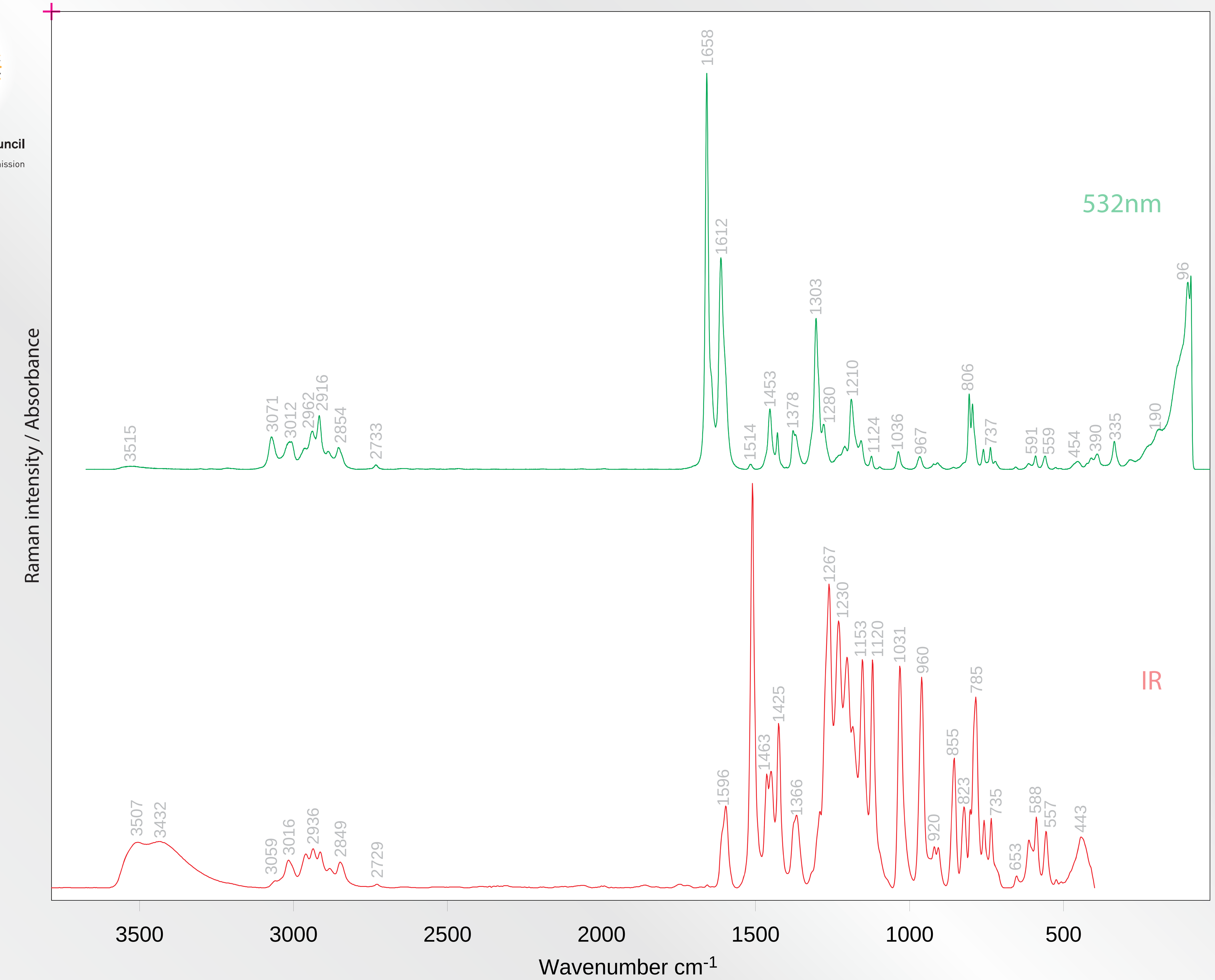

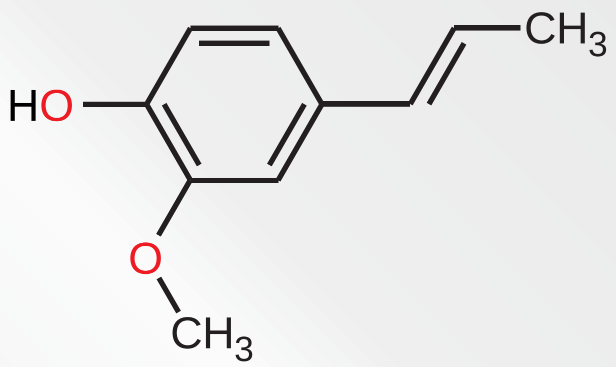

Sample:	I17206
Producer:	Sigma
Purity:	98%
State:	liquid
Temperature:	21.9°C
measured in:	-

Mode:	Vertex ATR
n_{Acc}:	32

λ_{ex}:	532.001 nm
LPO:	31.0 mW
ν (Si):	520.39 cm^{-1}
t_{int}:	1.09756 s
Grating:	1800 g/mm
p_{Laser}:	0°
$p_{Spectrometer}$:	unpol
Objective:	20x air NA 0.4

IR	532
3507	3515
3433	
	3071
3059	
3016	3012
	3006
2960	2962
2936	2938
2913	2916
2883	2886
2849	2854
2730	2733
	1658
	1612
1596	
1510	1514
1463	
1450	1453
1425	1429
	1378
1367	
	1303
1291	
	1280
1261	
1230	
1203	***1210***
1184	1189
1153	1158
1120	1124
	1096
1031	1036
960	967
920	922
906	909
855	858
823	826
803	***806***
	796
785	
758	760
735	737
	721
653	656
612	614
588	591
557	559
524	526
507	
	454
443	
	409
	390
	335
	283
	190
	96

Peter Bock
24.01.2018

ν...stretch, δ...in-plane bend, γ...out-of-plane bend, τ...torsion, γ_r...rocking, γ_w...wagging, γ_t...twisting Φ...ring (Varsanyi), d...degenerate
s...symmetric/scissoring, as... a(nti-)symmetric, ip...in-phase, op...out-of-phase

Bock and Gierlinger, J Raman Spectrosc. 2019; 1-15. https://doi.org/10.1002/jrs.5588

Cinnamaldehyde

European Research Council
Established by the European Commission
Grant No. 681885
www.bionami.at

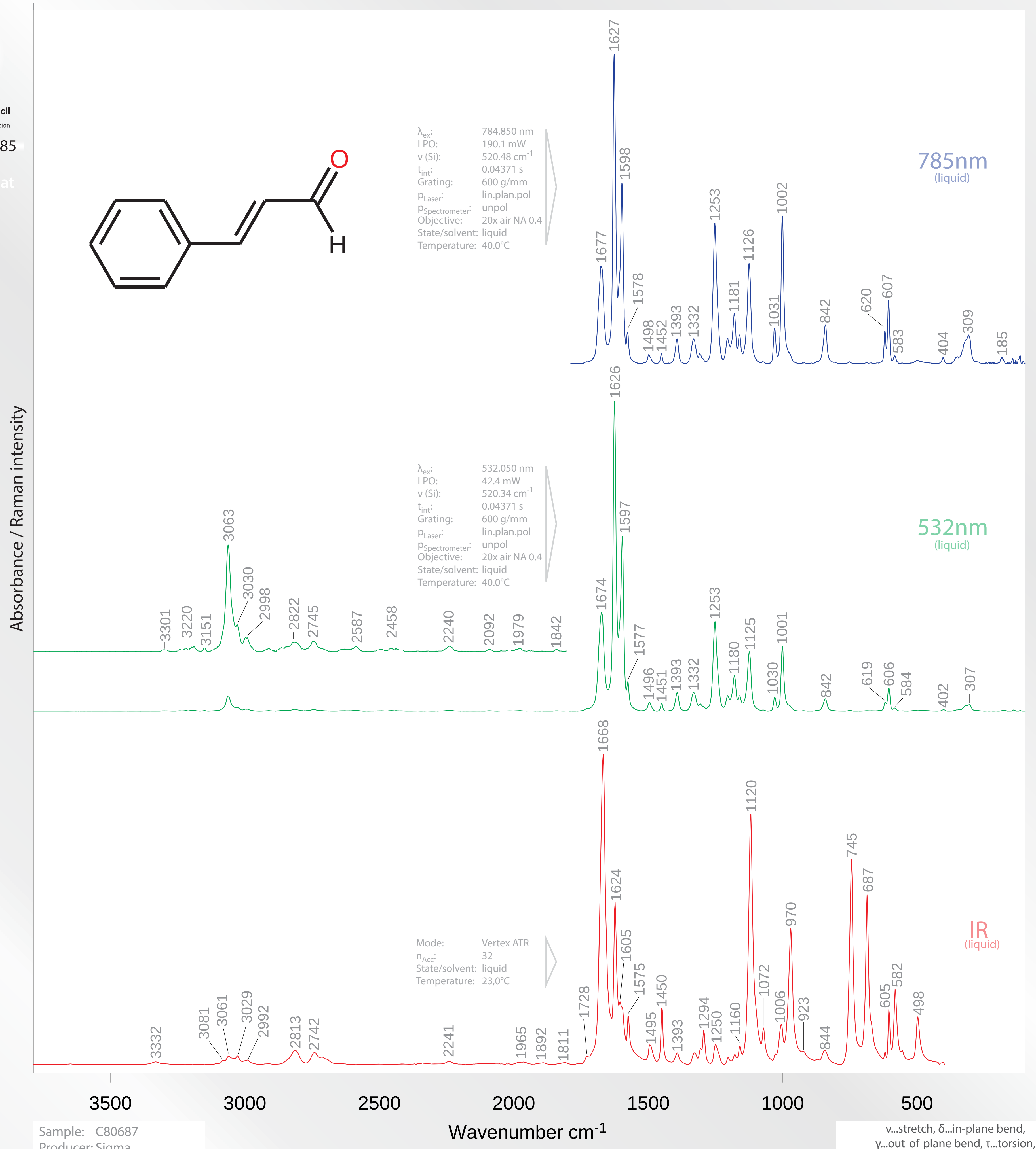

Sample: C80687
Producer: Sigma
Purity: 99%

785	532	IR	
		3332	2ν C=O (2 x 1668)
	3301	3300	ν C=O + ν C=C (1668 + 1626)
	3243		2ν C=C (2 x 1626)
	3220		ν C=C + ν C=C of ring Φ8a (1626 + 1597)
	3190		2ν C=C of ring Φ8a (2 x 1597)
	3151	3150	2ν C=C of ring Φ8a (2 x 1577)
		3104	
		3081	ν C-H of ring Φ20a
	3063	3061	ν C-H of ring Φ2
		3045	ν C-H of ring Φ7a
	3030	3029	ν C-H of ring Φ20b
	2998	2992	ν C-H of ring Φ13
	2912	2910	$ν_{as}$ C-H of C=C
	2865		
		2842	ν C-H of HC=O
	2822	2813	ν C-H of HC=O
	2745	2742	ν C-H of HC=O
		2714	ν C-H of HC=O
		2697	ν C-H of HC=O
	2637	2642	
	2587		
	2501		2ν $C_Φ$-C; 2δ C=C of ring Φ13
	2458		
	2240	2241	2ν C-C of C-C=O (2 x 1120)
	2092	2094	
	1979	1965	summation bands of γ C-H
	1842	1892	summation bands of γ C-H
		1811	summation bands of γ C-H
	1725	1728	ν C=O (impurity)
1677	1674	***1668***	ν C=O
1627	***1626***	1624	ν C=C
1598	***1597***	1605	ν C=C of ring Φ8a
1578	1577	1575	ν C=C of ring Φ8b
1498	1496	1495	ν C=C of ring Φ19a
1452	1451	1450	ν C=C of ring Φ19b
1393	1393	1393	δ C-H of HC=O
1332	1332	1328	δ C-H of sp^2-C; Φ3
1309	1307	1306	δ C-H of C=C; ν C=C of ring Φ14
1298		1294	
1253	1253	1250	ν $C_Φ$-C; δ C=C of ring Φ13▲
1205	1205	1203	
1181	1180	1178	δ C-H of ring Φ9a
1161	1161	1160	δ C-H of ring Φ15
1126	1125	***1120***	ν C-C of C-C=O
1075	1074	1072	δ C-H of ring Φ18b
1031	1030	1028	δ C-H of ring Φ18a
1002	1001	1006	δ C=C of ring Φ12△
977	975	970	γ C-H of C=C
		923	γ C-H of ring Φ17a
865		871	γ C-H of ring Φ10b
842	842	844	γ C-H of ring Φ10a (IR); ν $C_Φ$-C; ν C=C of ring Φ1 (Raman)▲
752		***745***	γ C-H of ring Φ11
		687	γ C=C of ring Φ4
620	619	620	δ C=C of ring Φ6b
607	606	605	δ C=C of ring Φ6a
583	584	582	δ C-C=O
		556	
500	497	498	γ C=C of ring Φ16b
404	402		γ C=C of ring Φ16a
350			
319			
309	307		
185	180		
145	141		
118			

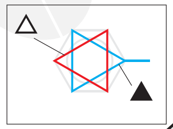

ν...stretch, δ...in-plane bend, γ...out-of-plane bend, τ...torsion, $γ_r$...rocking, $γ_w$...wagging, $γ_t$...twisting
Φ...ring (Varsanyi), d...degenerate
s...symmetric/scissoring, as... a(nti-)symmetric, ip...in-phase, op...out-of-phase

Peter Bock
14.01.2019

Bock and Gierlinger, J Raman Spectrosc. 2019; 1-15. https://doi.org/10.1002/jrs.5588

v2.2

European Research Council
Established by the European Commission
Grant No. 681885
www.bionami.at

Cinnamyl alcohol

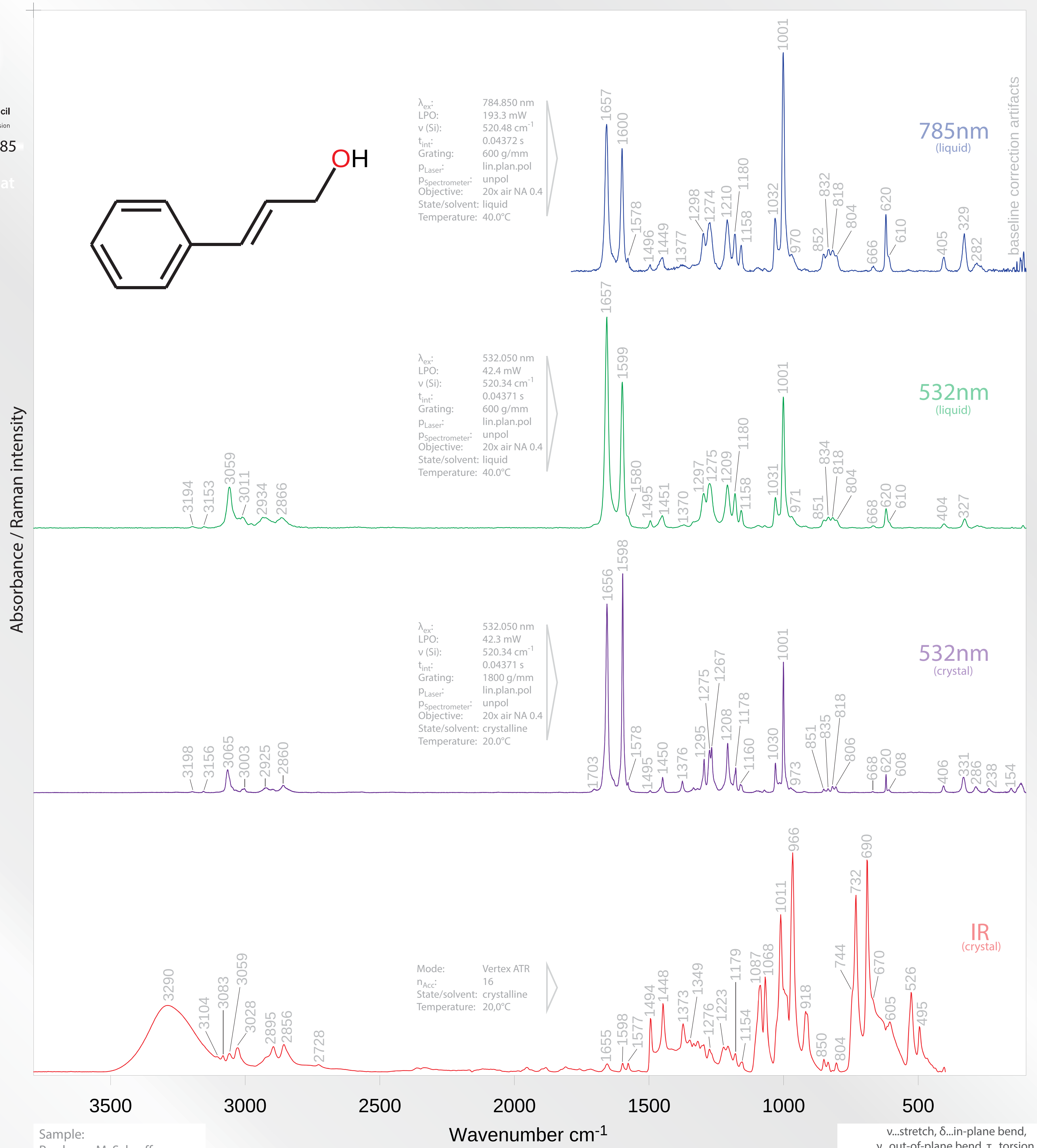

Sample:
Producer: M. Schroffenegger
Purity:

ν...stretch, δ...in-plane bend,
γ...out-of-plane bend, τ...torsion,
γ_r...rocking, γ_w...wagging, γ_t...twisting
Φ...ring (Varsanyi), d...degenerate
s...symmetric/scissoring, as... a(nti-)symmetric,
ip...in-phase, op...out-of-phase

785	532		IR	
			3290	ν O-H
	3194	3198		2ν C=C of ring Φ8a (2 x 1599)
	3153	3156		2ν C=C of ring Φ8b (2 x 1580)
			3104	
		3085	3083	ν C-H of ring Φ20a
	3059	3065	3059	ν C-H of ring Φ2
		3050		ν C-H of ring Φ7a
		3034	3028	ν C-H of ring Φ20b
	3011	3010		ν C-H of C=C
		3003		ν C-H of ring Φ13
	2978			
	2934	2925	2919	ν_{as} C-H of CH_2
		2896	2895	ν_{as} C-H of CH_2
	2866	2860	2856	ν_s C-H of CH_2
			2728	
		1703		
1657	***1657***	***1656***	1655	ν C=C
1600	***1599***	***1598***	1598	ν C=C of ring Φ8a
1578	1580	1578	1577	ν C=C of ring Φ8b
1496	1495	1495	1494	ν C=C of ring Φ19a
		1459		γ_w C-H of CH_2; δ O-H
1449	1451	1450	1448	ν C=C of ring Φ19b
1377	1370	1376	1373	
			1349	
1337	1335	1335	1334	δ C-H of ring Φ3; δ C-H of C=C
		1319	1319	
			1303	
1298	1297	1295	1297	ν C=C of ring Φ14; δ C-H of C=C
1274	1275	1275	1276	ν C=C of ring Φ14; δ C-H of C=C
		1267	1269	
1252				
			1223	δ O-H
1210	1209	1208	1207	ν $C_Φ$-C; δ C=C of ring Φ13 ▲
1180	1180	1178	1179	δ C-H of ring Φ9a
1158	1158	1160	1154	δ C-H of ring Φ15
		1101		
1095	1094	1088	1087	ν C-C; δ C-H of ring Φ18b
1073	1070	1072	1068	ν C-C; δ C-H of ring Φ18b
1032	1031	1030	1024	δ C-H of ring Φ18a
		1016	***1011***	ν C-O
1001	***1001***	***1001***	999	δ C=C of ring Φ12 △
			990	γ C-H of ring Φ5; γ_w C-H of C=C
970	971	973	***966***	γ_w C-H of C=C
921	922	924	918	γ C-H of ring Φ17a; ν_s C-C-O
			913	γ C-H of ring Φ17a; ν_s C-C-O
852	851	851	850	γ C-H of ring Φ10b
832	834	835	835	γ C-H of ring Φ10a
818	818	818	818	
804	804	806	804	ν $C_Φ$-C; ν C=C of ring Φ1 ▲
			744	
			732	γ C-H of ring Φ11
			690	γ C=C of ring Φ4
666	668	668	670	
			637	
620	620	620	615	δ C=C of ring Φ6b
610	608	608	605	δ C=C of ring Φ6a
537		526	526	γ C=C of ring Φ16b
		496	495	
			468	
			438	
405	404	406		γ C=C of ring Φ16a
329	327	331		
282	280	286		
		238		
		154		

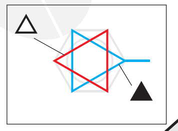

Peter Bock
08.01.2019

Bock and Gierlinger, J Raman Spectrosc. 2019; 1-15. https://doi.org/10.1002/jrs.5588

v2.2

Hydrocinnamaldehyde

European Research Council
Established by the European Commission
Grant No. 681885
www.bionami.at

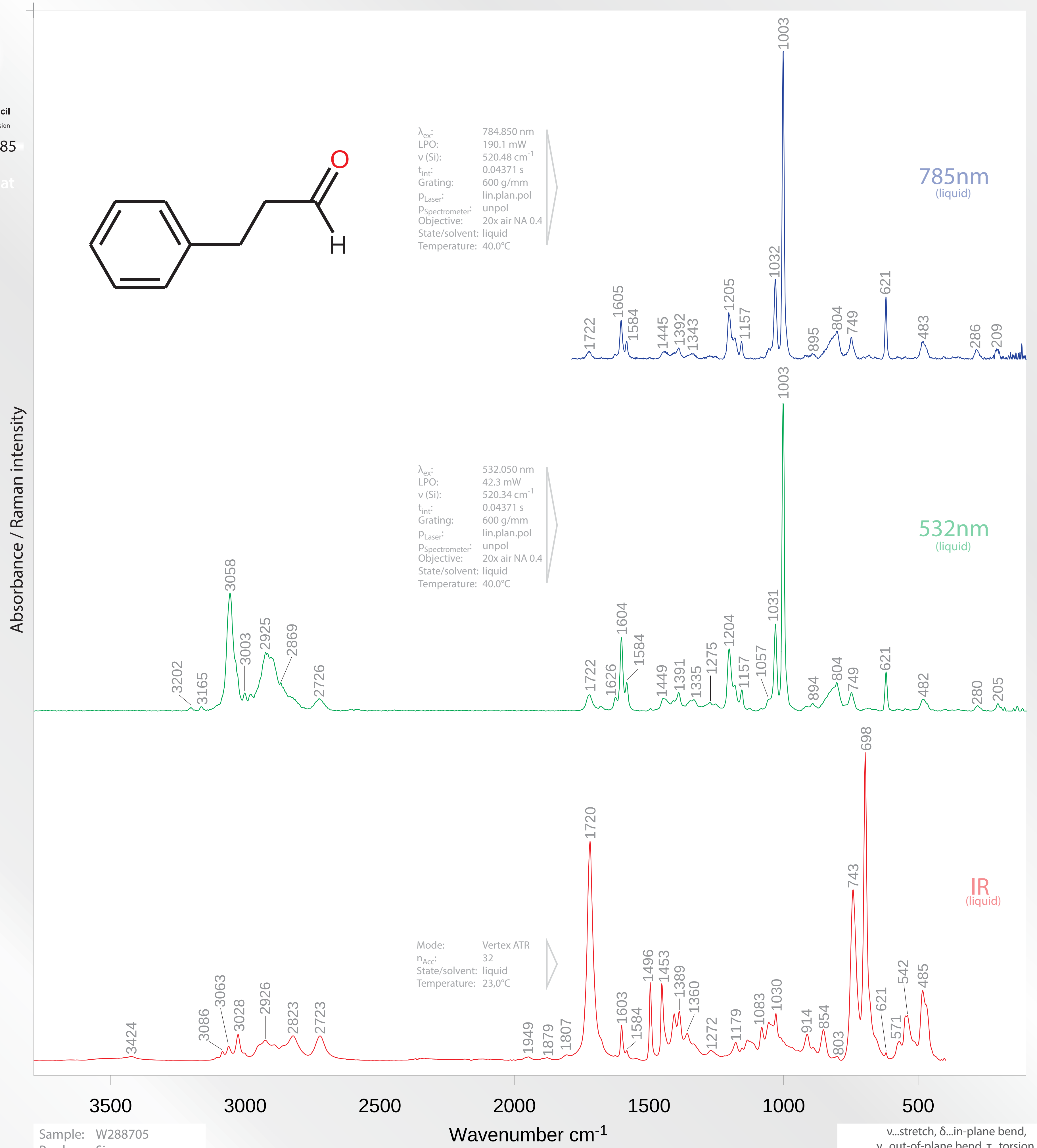

Sample: W288705
Producer: Sigma
Purity: 95%

ν...stretch, δ...in-plane bend, γ...out-of-plane bend, τ...torsion, γr...rocking, γw...wagging, γt...twisting, Φ...ring (Varsanyi), d...degenerate, s...symmetric/scissoring, as... a(nti-)symmetric, ip...in-phase, op...out-of-phase

785	532	IR	
		3424	2ν C=O (2 x 1720)
	3202		2ν C=C of ring Φ8a (2 x 1604)
	3165		2ν C=C of ring Φ8b (2 x 1584)
		3106	
		3086	ν C-H of ring Φ20a
	3058	3063	ν C-H of ring Φ2
	3038		ν C-H of ring Φ7a
	3030	3028	ν C-H of ring Φ20b
	3003	3005	ν C-H of ring Φ13
	2980		ν_{as} C-H of CH_2
		2945	ν_{as} C-H of CH_2
	2925	2926	ν_{as} C-H of CH_2
	2905	2893	ν_s C-H of CH_2
	2869	2856	ν_s C-H of CH_2
		2823	ν C-H of HC=O
	2726	2723	ν C-H of HC=O
		1949	summation bands of γ C-H
		1879	summation bands of γ C-H
		1807	summation bands of γ C-H
1722	1722	***1720***	ν C=O
	1681		cinnamaldehyde impurity
1629	1626	1629	cinnamaldehyde impurity
1605	***1604***	1603	ν C=C of ring Φ8a
1584	1584	1584	ν C=C of ring Φ8b
		1548	
1499	1496	1496	ν C=C of ring Φ19a
1445	1449	1453	ν C=C of ring Φ19b
	1411	1408	δ_s C-H of CH_2
1392	1391	1389	δ C-H of HC=O
	1350	1360	γ_w C-H of CH_2
1343	1335	1336	δ C-H of ring Φ3
1275	1275	1272	ν C=C of ring Φ14;
1252	1254		γ_w C-H of CH_2
1205	***1204***		ν C_Φ-C; δ C=C of ring Φ13 ▲
1193			ν C_Φ-C; δ C=C of ring Φ13 ▲
1182	1182	1179	δ C-H of ring Φ9a
1157	1157	1155	δ C-H of ring Φ15
	1127	1136	γ_t C-H of CH_2
		1116	
1088	1084	1083	ν C-C; δ C-H of ring Φ18b
1057	1057	1056	ν C-C; δ C-H of ring Φ18b
1032	***1031***	1030	δ C-H of ring Φ18a
		1013	ν C-C
1003	***1003***	1001	δ C=C of ring Φ12 △
		986	γ C-H of ring Φ5
	917	971	
918		914	ν C-C; γ C-H of ring Φ17a
895	894	892	γ_r C-H of CH_2; γ C-H of HC=O
		854	γ C-H of ring Φ10b
804	804	803	ν C_Φ-C; ν C=C of ring Φ1 ▲
749	749	***743***	γ C-H of ring Φ11
685	684	***698***	γ C=C of ring Φ4
662		659	γ_r C-H of CH_2; γ C-H of HC=O
621	621	621	δ C=C of ring Φ6b
577	579	571	δ C=C of ring Φ6a
550	549	549	δ C-C=O
		542	δ C-C=O
483	482	485	γ C=C of ring Φ16b
		472	
437			
408			γ C=C of ring Φ16a
374			
354			
286	280		
209	205		

Peter Bock 27.12.2018

Bock and Gierlinger, J Raman Spectrosc. 2019; 1-15. https://doi.org/10.1002/jrs.5588

3-Phenyl-1-propanol

European Research Council
Established by the European Commission
Grant No. 681885
www.bionami.at

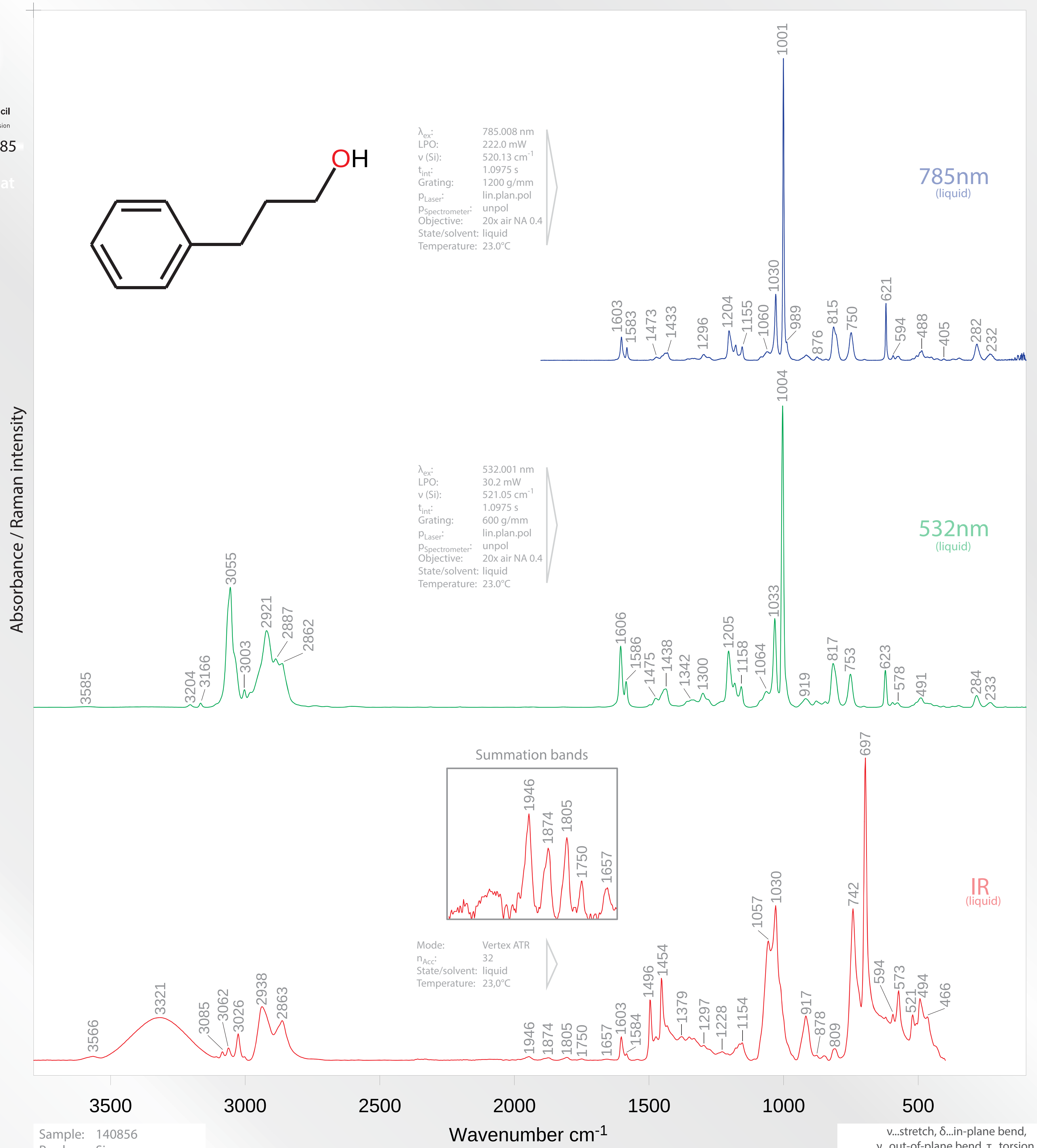

Sample: 140856
Producer: Sigma
Purity: 98%

ν...stretch, δ...in-plane bend,
γ...out-of-plane bend, τ...torsion,
γ_r...rocking, γ_w...wagging, γ_t...twisting
Φ...ring (Varsanyi), d...degenerate
s...symmetric/scissoring, as... a(nti-)symmetric,
ip...in-phase, op...out-of-phase

785	532	IR	
	3585	3566	ν O-H
		3321	ν O-H
	3204		2ν C=C of ring Φ8a (2 x 1606)
	3166		2ν C=C of ring Φ8b (2 x 1586)
	3110	3107	
		3085	ν C-H of ring Φ20a
	3055	3062	ν C-H of ring Φ2
	3040		ν C-H of ring Φ7a
		3026	ν C-H of ring Φ20b
	3003	3003	ν C-H of ring Φ13
	2980		$\nu_{ip, as}$ C-H of CH_2
	2949	2938	$\nu_{op, as}$ C-H of CH_2
	2921		ν C-H
	2887	2878	$\nu_{ip, s}$ C-H of CH_2
	2862	2863	$\nu_{op, s}$ C-H of CH_2
		1946	summation bands of γ C-H
		1874	summation bands of γ C-H
		1805	summation bands of γ C-H
		1750	summation bands of γ C-H
		1657	summation bands of γ C-H
1603	1606	1603	ν C=C of ring Φ8a
1583	1586	1584	ν C=C of ring Φ8b
1496	1498	1496	ν C=C of ring Φ19a
1473	1475	1474	δ_s C-H of CH_2
1452		1454	ν C=C of ring Φ19b
1440	1438	1433	δ_s C-H of CH_2
1433			δ_s C-H of CH_2
		1379	γ_w C-H of CH_2; δ O-H
1358	1357	1351	γ_w C-H of CH_2; δ O-H
1342			γ_w C-H of CH_2; δ O-H
1332	1335	1333	δ C-H of ring Φ3
1296	1300	1297	ν C=C of ring Φ14;
1275	1280	1273	
1230	1236	1228	
1204	1205	1209	ν $C_Φ$-C; δ C=C of ring Φ13 ▲
1178	1182	1178	δ C-H of ring Φ9a
		1164	γ_t C-H of CH_2; δ C-H of ring Φ15
1155	1158	1154	γ_t C-H of CH_2; δ C-H of ring Φ15
		1110	
1084	1089	1085	γ_t C-H of CH_2; δ C-H of ring Φ18b
1060	1064	***1057***	ν C-C; δ C-H of ring Φ18b
1030	***1033***	***1030***	δ C-H of ring Φ18a
		1012	ν C-C; ν C-O
1001	***1004***	1001	δ C=C of ring Φ12 △
989		983	ν C-C; γ C-H of ring Φ5
917	919	917	ν C-O; ν C-C; γ C-H of ring Φ17a
876	879	878	γ_r C-H of CH_2
866			γ C-H of ring Φ10b
844	846	850	γ C-H of ring Φ10a
815	817	814	ν $C_Φ$-C; ν C=C of ring Φ1▲
808		809	ν $C_Φ$-C; ν C=C of ring Φ1▲
750	753	***742***	γ C-H of ring Φ11
700	703	***697***	γ C=C of ring Φ4
621	623	621	δ C=C of ring Φ6b
594	596	594	δ C=C of ring Φ6a
575	578	573	
522		521	γ C=C of ring Φ16b
507	509	507	
494	491	494	
488			
467	470	466	
453	456		
430	434	434	
405	407		γ C=C of ring Φ16a
372	352		

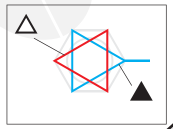

Peter Bock
09.01.2019

Bock and Gierlinger, J Raman Spectrosc. 2019; 1-15. https://doi.org/10.1002/jrs.5588
